# Post-stroke Cognitive Impairment—Impact of Follow-Up Time and Stroke Subtype on Severity and Cognitive Profile: The Nor-COAST Study

**DOI:** 10.3389/fneur.2020.00699

**Published:** 2020-07-17

**Authors:** Stina Aam, Marte Stine Einstad, Ragnhild Munthe-Kaas, Stian Lydersen, Hege Ihle-Hansen, Anne-Brita Knapskog, Hanne Ellekjær, Yngve Seljeseth, Ingvild Saltvedt

**Affiliations:** ^1^Department of Neuromedicine and Movement Science, Faculty of Medicine and Health Science, NTNU-Norwegian University of Science and Technology, Trondheim, Norway; ^2^Department of Geriatric Medicine, Clinic of Medicine, St. Olavs Hospital, Trondheim University Hospital, Trondheim, Norway; ^3^Department of Medicine, Vestre Viken Hospital Trust, Bærum Hospital, Drammen, Norway; ^4^Institute of Clinical Medicine, University of Oslo, Oslo, Norway; ^5^Department of Mental Health, Faculty of Medicine and Health Science, NTNU-Norwegian University of Science and Technology, Trondheim, Norway; ^6^Department of Geriatric Medicine, Oslo University Hospital, Oslo, Norway; ^7^Stroke Unit, Department of Internal Medicine, St. Olavs Hospital, Trondheim University Hospital, Trondheim, Norway; ^8^Medical Department, Ålesund Hospital, Møre and Romsdal Health Trust, Ålesund, Norway

**Keywords:** post-stroke cognitive impairment, vascular dementia, stroke, stroke subtype, cognitive domains, cerebrovascular disease, intracerebral hemorrhag, prognosis

## Abstract

**Background:** Post-stroke cognitive impairment (PSCI) is common, but evidence of cognitive symptom profiles, course over time, and pathogenesis is scarce. We investigated the significance of time and etiologic stroke subtype for the probability of PSCI, severity, and cognitive profile.

**Methods:** Stroke survivors (*n* = 617) underwent cognitive assessments of attention, executive function, memory, language, perceptual-motor function, and the Montreal Cognitive Assessment (MoCA) after 3 and/or 18 months. PSCI was classified according to DSM-5 criteria. Stroke severity was assessed with the National Institutes of Health Stroke Scale (NIHSS). Stroke subtype was categorized as intracerebral hemorrhage (ICH), large artery disease (LAD), cardioembolic stroke (CE), small vessel disease (SVD), or un-/other determined strokes (UD). Mixed-effects logistic or linear regression was applied with PSCI, MoCA, and z-scores of the cognitive domains as dependent variables. Independent variables were time as well as stroke subtype, time, and interaction between these. The analyses were adjusted for age, education, and sex. The effects of time and stroke subtype were analyzed by likelihood ratio tests (LR).

**Results:** Mean age was 72 years (SD 12), 42% were females, and mean NIHSS score at admittance was 3.8 (SD 4.8). Probability (95% CI) for PSCI after 3 and 18 months was 0.59 (0.51–0.66) and 0.51 (0.52–0.60), respectively and remained constant over time. Global measures and most cognitive domains were assessed as impaired for the entire stroke population and for most stroke subtypes. Executive function and language improved for the entire stroke population (LR) = 9.05, *p* = 0.003, and LR = 10.38, *p* = 0.001, respectively). After dividing the sample according to stroke subtypes, language improved for ICH patients (LR = 18.02, *p* = 0.003). No significant differences were found in the severity of impairment between stroke subtypes except for attention, which was impaired for LAD and CE in contrast to no impairment for SVD (LR = 56.58, *p* < 0.001).

**Conclusions:** In this study including mainly minor strokes, PSCI is common for all subtypes, both early and long-term after stroke, while executive function and language improve over time. The findings might contribute to personalizing follow-up and offer new insights into underlying mechanisms. Further research is needed on underlying mechanisms, PSCI prevention and treatment, and relevance for rehabilitation.

## Introduction

Stroke is one of two leading causes of disability-adjusted life-years worldwide (1), and post-stroke cognitive impairment (PSCI) has been shown to be common among stroke survivors. Recent reviews and meta-analyses identified a pooled prevalence of PSCI of 53.4% and mild and major PSCI of 36.4–38 and 16% respectively, measured within 1.5 years post-stroke (2, 3).

Previous studies have reported conflicting results regarding the prognosis for patients suffering PSCI; these have indicated deterioration, no progression, and even improvement in cognition over time for subgroups (4–11). Several cognitive domains are affected in PSCI; of these, impairment in attention and executive function seem to be the most prevalent and severe shortly after and a long time after suffering a stroke (12–16). A recent study on PSCI a short time after a stroke showed a high prevalence of impairment in global cognition and in the five most commonly assessed domains: attention, memory, language, perceptual-motor function, and executive function (17).

The underlying pathological mechanisms for suffering a stroke are heterogeneous, and severity and localization of the stroke are important for PSCI (6, 17, 18). About 10–20% of strokes are hemorrhagic; the rest are ischemic and typically related to large artery disease (LAD), cardioembolic stroke (CE), or small vessel disease (SVD), often labeled lacunar infarction, with about 25% in each category (19–21). LAD and CE strokes are often cortical strokes of large volume, while SVD strokes are subcortical and of small volume (22). Cognitive impairment has been shown to be less common in the early post-stroke period in SVD compared to other stroke subtypes, but SVD is associated with cognitive decline long after a stroke (16, 17, 23, 24). However, in their review and meta-analyses, Makin et al. found similar proportions to have PSCI in lacunar vs. non-lacunar stroke [OR 0.75 (95% CI 0.47–1.20)] (25, 26). ICH has been reported to be more strongly associated with dementia than ischemic stroke (6), and impairments in processing speed, executive function, episodic memory, language, and visuo-spatial abilities have been found to be most prevalent (19, 21, 27).

There remains a need for additional knowledge about the course of PSCI and the impact of stroke subtypes on PSCI. Therefore, the aim of this study was to investigate whether time and etiological stroke subtype impact the probability for PSCI and its severity and cognitive symptom profile three and 18 months post-stroke.

## Methods

The present study is part of the Norwegian Cognitive Impairment After Stroke (Nor-COAST) study, a multicenter prospective cohort study that recruited participants hospitalized with acute stroke in five Norwegian stroke units from May 2015 through March 2017 (28). Inclusion criteria were hospitalization with acute ischemic or hemorrhagic stroke within one week after symptom appearance, fluency in a Scandinavian language, and age over 18 years. The only exclusion criterion was expected survival <3 months. Participation in the study was voluntary, and the participants gave informed written consent. When a person was unable to give consent, informed written consent for participation was given by a proxy family member. The study was approved by the Regional Committee for Medical and Health Research Ethics (REC Nord 2015/171) and registered in ClinicalTrials.gov (NCT02650531). Further details are described in the protocol for the Nor-COAST study (28).

### Clinical Assessments

Data on demographic characteristics and vascular risk factors were collected from medical records. Vascular risk factors were defined as described in previous work in the Nor-COAST study (29) and in the Supplementary Material. Stroke severity was assessed with the National Institutes of Health Stroke Scale (NIHSS) (30). Ischemic strokes were classified according to the Trial of Org 10172 in Acute Stroke Treatment (TOAST) classification (22) by experienced stroke physicians. Further stepwise classification into TOAST modified was done as described in the Supplementary Material and Supplementary Figure S1. Stroke subtype was defined by ICH and modified TOAST classification into large artery disease (LAD), cardioembolic stroke (CE), small vessel disease (SVD), other etiology, and undetermined strokes; as the subtype other etiology comprised a small number, it was grouped with undetermined etiology (UD). Localization of symptoms in the acute phase was collected at admission and categorized as right, left, bilateral, or unable to locate by side.

### Cognitive and Functional Assessments

Cognitive function was assessed by trained study staff with a cognitive test battery recommended by the National Institute of Neurological Disorders–Canadian Stroke Network (NINDS–CSN) Harmonization Standards (31) adjusted to available and validated cognitive tests in Norwegian. The test battery comprised the Word List Memory and Recall Test and Verbal Fluency Test Category (animals) from the Consortium to Establish a Registry for Alzheimer's Disease (CERAD) battery (32, 33); Verbal Fluency Test Letter (FAS) (34, 35); Trail Making Tests A (TMT-A) and B (TMT-B) (36); and the Montreal Cognitive Assessment (MoCA) (37), version 7.3 at the 3-months follow-up and version 7.1 at the 18-months follow-up. Cognitive function was also assessed with the Global Deterioration Scale (GDS), a global measure of cognitive function originally designed to measure cognitive decline in Alzheimer's disease but shown to be valid also in measuring vascular dementia (29, 38–40). Activities of daily living (ADL) were assessed with the Barthel Index (BI) (41) and functional outcome with the Modified Rankin Scale (mRS) (42). Baseline assessments were performed during hospital stays. Follow-ups at 3 and 18 months were performed at the hospitals' outpatient clinics. For participants unable to attend, assessments were performed through telephone interviews with the participants, their caregivers, or nursing home staff with the mRS, BI, GDS, and if possible, the Telephone MoCA (T-MoCA) (43).

### Outcomes

Cognitive outcome assessments included complex attention measured by TMT-A, executive function by TMT-B and Verbal Fluency Test Letters (FAS), memory by Word List Recall, language by Verbal Fluency Test Category (animals), and perceptual-motor function by the visuospatial/executive part of MoCA. Cognitive status was dichotomized into normal cognition and cognitive impairment; cognitive impairment comprised both mild and major neurocognitive disorders (NCD), and the cut-off for cognitive impairment was defined according to the cut-off for mild NCD in the 5th edition of the Diagnostic and Statistical Manual of Mental Disorders (DSM-5) criteria for mild and major neurocognitive disorders (44). Details are described in previous work in the Nor-COAST study (29) and summarized in the Supplementary Material.

### Statistics

Z-scores normalized by mean and SD of the normative data were derived from the raw scores of the cognitive tests. The normative data used are presented in previous work (29) as well as in Supplementary Table S1. The symptom profile of PSCI was measured by the z-scores of the five cognitive domains. The cognitive domains were measured by the z-score of the single completed cognitive test. Two tests were administered to measure executive function, and the average z-score was used. The z-scores were implemented with lower z-scores indicating poorer outcomes. The severity of PSCI was measured by z-scores of global z and MoCA; global z was defined as the average scores of the five cognitive domains assessed.

To minimize bias from excluded participants, imputation was performed as described in previous work (29) and as described in the Supplementary Material.

Probability for PSCI and severity and symptom profile of PSCI were analyzed as appropriate with mixed-effects logistic or linear regression with PSCI according to DSM-5 criteria, MoCA, and global z and z-scores for the five cognitive domains of attention, executive function, memory, language, and perceptual-motor function as dependent variables one at a time. The independent variables were time (model 1), stroke subtype, time and the interaction between stroke subtype and time (model 2), and stroke subtype (model 3). We adjusted for age, education, and sex. The estimated probability for PSCI according to DSM-5 criteria was calculated from the estimated odds in mixed-effects logistic regression, as probability = odds (1+odds). Mixed-effects logistic and linear regression models were preferred since a mixed-effects linear regression model minimizes bias by handling missing data in an appropriate way under a missing at random assumption, and also because mixed-effects logistic regression models with categorical time effects often produce fairly robust estimates in a mild departure from data missing completely at random (45). Illustrations of the statistical models for the logistic and linear regressions are provided in Supplementary Figures S2, S3. Hypothesis tests for the effects of time and stroke subtype in model 2 were conducted by likelihood ratio tests comparing model 1 and model 2, as well as comparing model 2 and model 3. The results were presented as estimates with mean and 95% confidence intervals (CI) and the test statistics with degrees of freedom and *p*-value.

Sensitivity analyses with the exclusion of participants deceased at 18 months (*n* = 21), as well as with the exclusion of pre-stroke dementia defined as pre-stroke GDS 4–7 (*n* = 23), were performed to determine if these affected the outcome. We also performed sensitivity analyses for unadjusted analyses; analyses adjusted for age, education, sex, pre-stroke mRS, and NIHSS combined; and analyses adjusted for age, education, sex, and location of symptoms combined.

PSCI according to DSM-5 criteria, stroke subtype, time, and sex were analyzed as categorical variables, while global z, MoCA z-score, z-scores of the cognitive domains, age, education, mRS, and NIHSS were analyzed as continuous variables. Complete case analyses were used for stroke subtype, age, education, and sex, while available case analyses were used for PSCI according to DSM-5 criteria, global z, MoCA, z-scores for the cognitive domains, mRS, and NIHSS. Confounders were included as fixed effects, while subject and hospital were included as random effects.

Due to multiple hypotheses, we considered two-tailed *p* < 0.01 as statistically significant. Data were analyzed using SPSS 25 and STATA 16.0.

## Results

### Baseline Characteristics

**Figure 1:**
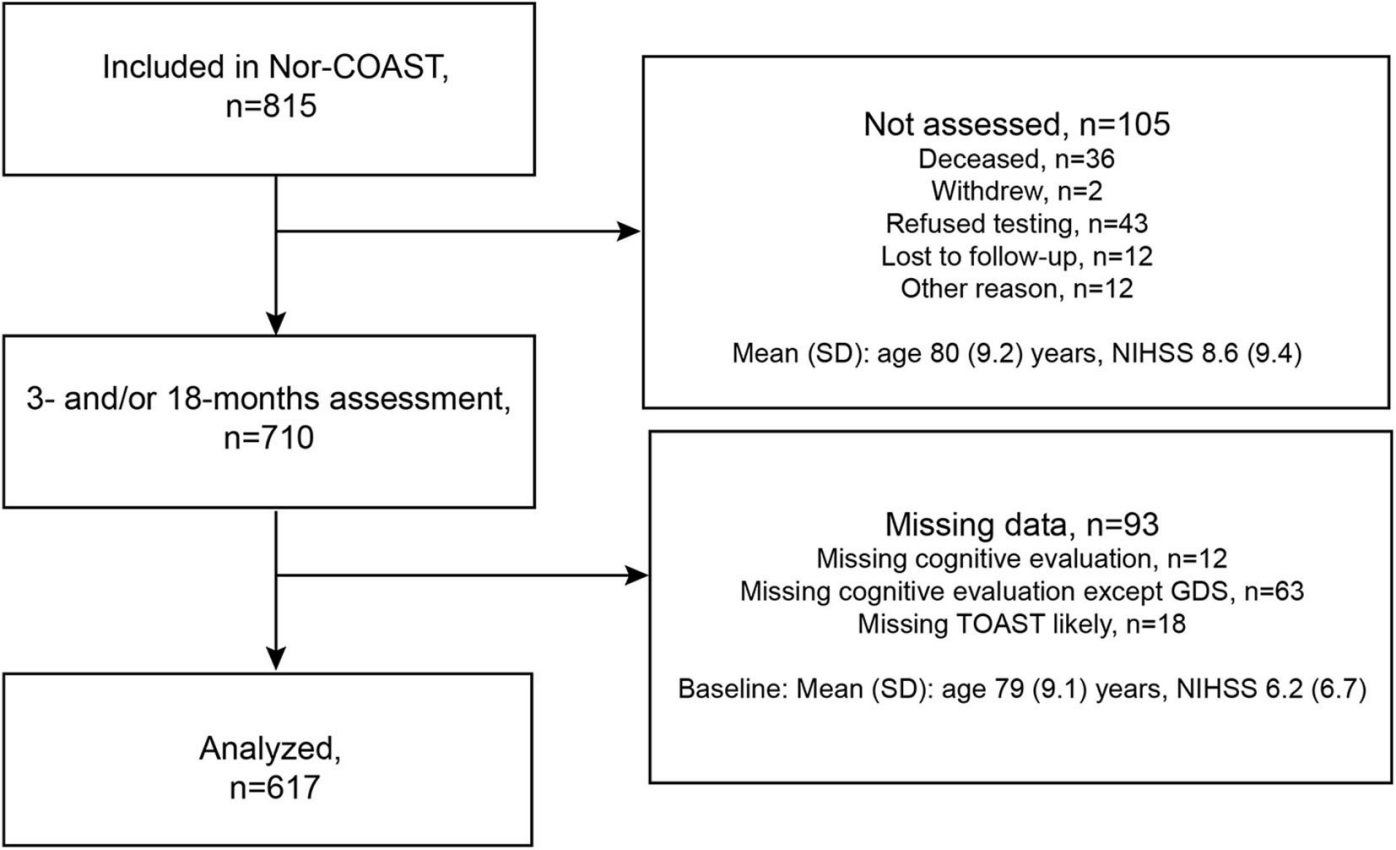
Flowchart of participants included in the study.

Of the 815 participants included in the Nor-COAST study, 700 were assessed at 3 months and 599 at the 18-months follow-up, 10 of whom were not assessed at 3 months. Of the 710 participants assessed at either 3 or 18 months, 93 were excluded due to missing data, resulting in a study sample of 617 participants (Figure 1). Of these 617 participants, 21 were deceased at 18 months.

The mean age was 72 years (SD 12), 42% were females, the mean education was 12.5 years (SD 3.8), and the mean NIHSS score at admittance was 3.8 (SD 4.8). The baseline characteristics of the participants are shown in Table 1. The 198 participants excluded were age 80 years (SD 9.1); 55% were females; mean years of education were 10.5 years (SD 3.1); and mean NIHSS score at admittance was 7.4 (8.2). Among 192 of those excluded, 36 (19%) had a pre-stroke GDS of 1 (mild NCD) and 38 (20%) had a pre-stroke GDS of 4–7 (major NCD).

**Table 1:** Baseline characteristics.

**Demographics**	***N* = 617**		
Mean age, years (SD)		72	(12)
Male sex, *n* (%)		360	(58)
Mean education, years (SD)		12.5	(3.8)
**Vascular risk factors**, ***n*** **(%)**			
Hypertension, *n* (%)	*N* = 617	338	(55)
Hypercholesterolemia, *n* (%)	*N* = 617	314	(46)
Current cigarette smoking, *n* (%)	*N* = 615	119	(19)
Diabetes mellitus, *n* (%)	*N* = 617	115	(19)
Mean BMI, kg/m^2^ (SD)	*N* = 583	26.1	(4.1)
**Vascular disease**, ***n*** **(%)**	*N* = 617		
Coronary heart disease, *n* (%)		108	(18)
Atrial fibrillation, *n* (%)		144	(23)
Previous stroke or TIA, *n* (%)		136	(22)
**Stroke subtype**, ***n*** **(%)**	*N* = 618		
Cerebral infarction		564	(91)
Cerebral hemorrhage		53	(8.6)
TOAST classification^*^, ***n*** **(%)**	*N* = 564		
Large vessel disease		140	(25)
Cardioembolic disease		153	(27)
Small vessel disease		135	(24)
Other etiology		17	(3.0)
Undetermined etiology		119	(21)
**Symptom locations**, ***n*** **(%)**	*N* = 599		
Right		243	(41)
Left		272	(45)
Bilateral		18	(3.0)
Not able to locate by side		66	(11)
**Thrombolysis**, ***n*** **(%)**	*N* = 612	147	(24)
**Thrombectomy**, ***n*** **(%)**	*N* = 617	12	(1.9)
**Pre-stroke GDS (1-7)**, ***n*** **(%)**	*N* = 611		
GDS = 1–2 (Normal cognition)		553	(91)
GDS = 3 (Mild Neurocognitive Disorder)		35	(5.7)
GDS = 4–7 (Major Neurocognitive Disorder)		23	(3.8)
**Assessments**			
NIHSS (0–42) at admittance, mean (SD)	*N* = 601	3.8	(4.8)
Pre-stroke mRS (0–6), mean (SD)	*N* = 613	0.77	(1.0)
mRS (0–6) at discharge, ^†^ mean (SD)	*N* = 615	2.1	(1.3)
Barthel Index (0–100) at discharge, ^†^ mean (SD)	N = 615	89	(19)
MoCA total score (0–30) during hospital stay, mean (SD)	*N* = 575	24	(4.8)

*SD, Standard Deviation; BMI, Body Mass Index; TIA, Transient Ischemic Attack; TOAST, Trial of Org 10172 in Acute Stroke Treatment; GDS, Global Deterioration Scale; NIHSS, National Institutes of Health Stroke Scale; mRS, modified Rankin Scale*.

* *TOAST modification; Undetermined etiology of TOAST probable, based on original classification (22); first classified as TOAST possible, also based on original classification (22); then as TOAST likely (47) where participants with findings of carotid stenosis <50% were classified as large artery disease. Finally, TOAST modified was developed by merging TOAST probable, TOAST possible, and TOAST likely*.

† *at discharge or day 7 if length of stay extends beyond 7 days*.

The numbers of participants completing cognitive tests for the cognitive domains, with mean z-scores of the tests and proportions with z-scores <-1.5, are shown in Supplementary Table S2.

### Probability for PSCI and Impairments in the Cognitive Domains at 3 and 18 Months

**Figure 2:**
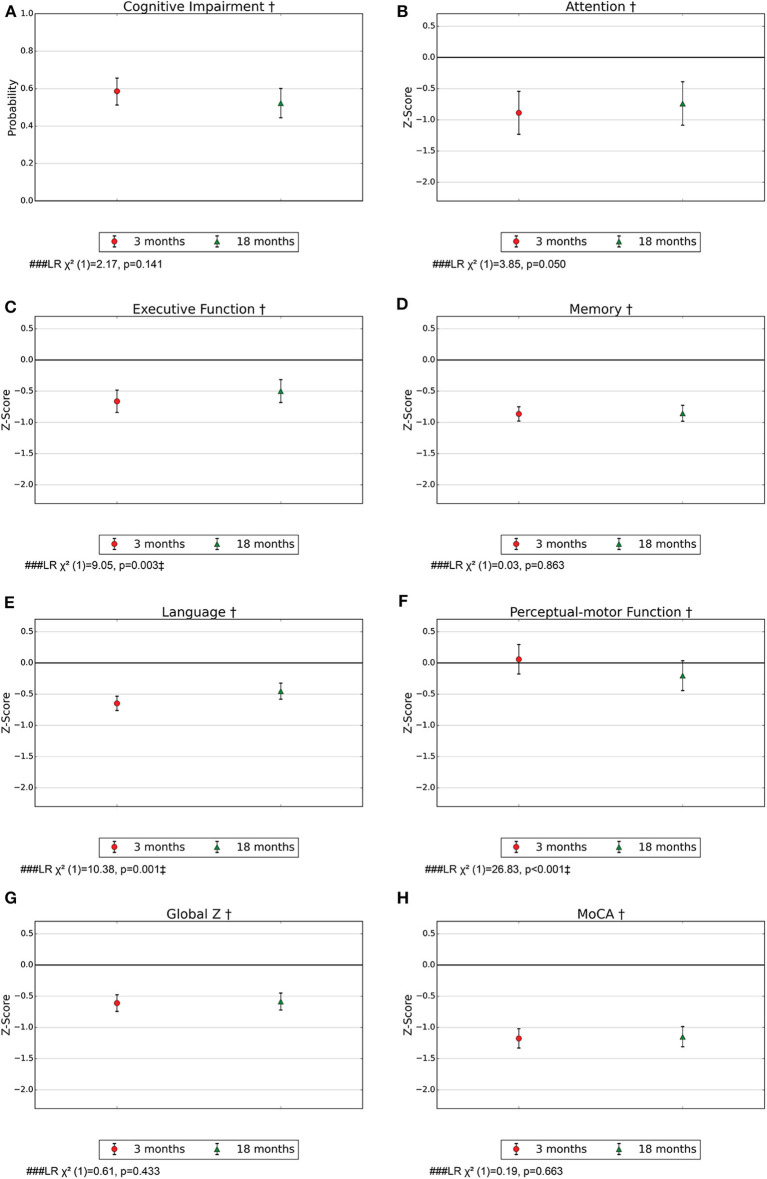
**(A–H)** Probability for cognitive impairment according to DSM-5 criteria with 95% CI and mean z-score with 95% CI for the cognitive domains at 3 and 18 months post-stroke in model 1. MoCA, Montreal Cognitive Assessment. †Adjusted for age, education, and sex. ^*###*^ LR χ^2^(1), Likelihood Ratio test model 1 vs a model with only age, education, and sex as confounders, with one degree of freedom; hypothesis test of whether there is an effect of time. ^‡^*p* < 0.01.

**Figure 3:**
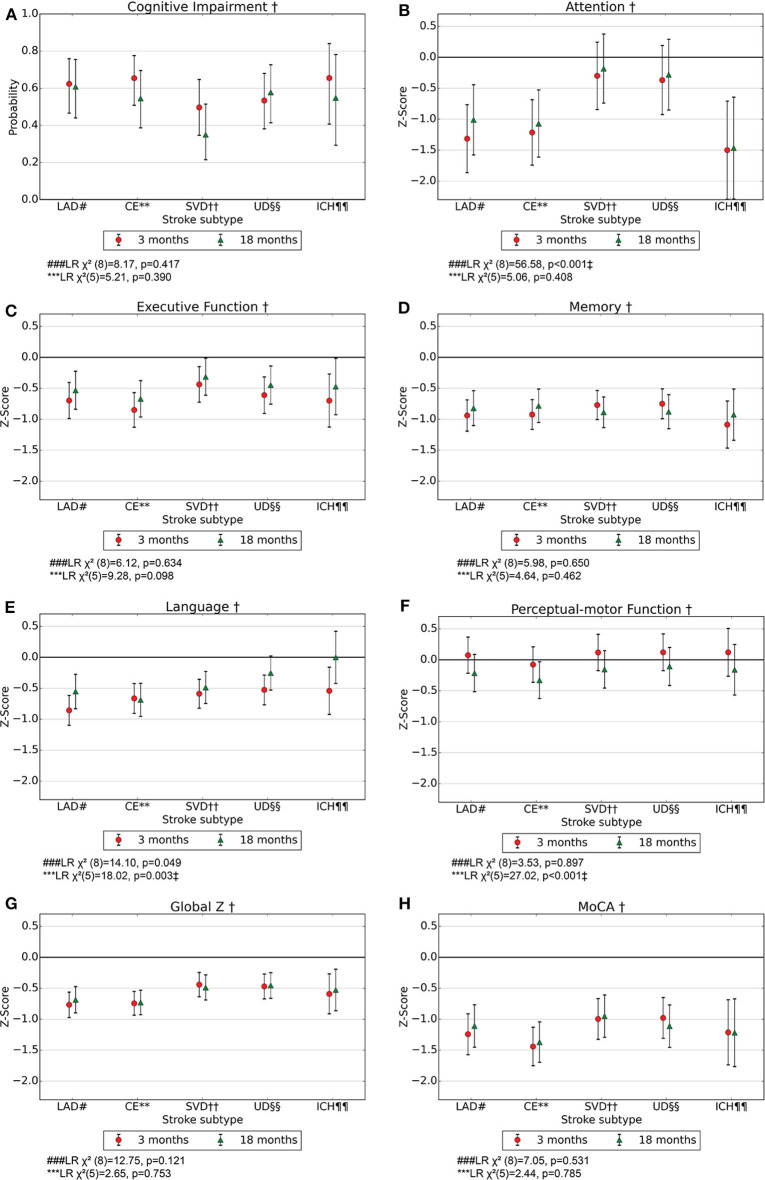
**(A–H)** Probability for cognitive impairment according to DSM-5 criteria with 95% CI and mean z-score with 95% CI for the cognitive domains at 3- and 18-months post-stroke in model 2. MoCA, Montreal Cognitive Assessment. † Adjusted for age, education, and sex. ^#^LAD, Large artery disease. **CE, Cardiac emboli. ††SVD, Small vessel disease. ^§§^UD, Undetermined and other determined strokes. ^¶¶^ICH, Intracerebral hemorrhage. ^*###*^LR χ^2^(8), Likelihood Ratio test model 1 vs. model 2 with 8 degrees of freedom; hypothesis test of whether there is an effect of stroke subtype. ***LR χ^2^(5), Likelihood Ratio test model 2 vs. model 3 with 5 degrees of freedom; hypothesis test of whether there is an effect of time for at least one stroke subtype. ^‡^*p* < 0.01.

For the entire study population, the probability for PSCI according to DSM-5 criteria was 0.59 (95% CI 0.51–0.65) after 3 months and 0.51 (95% CI 0.52–0.60) after 18 months (Figure 2). For the different stroke subtypes, the probability for PSCI at 3 months ranged from 0.50 (95% CI 0.35–0.65) for SVD to 0.66 (95% CI 0.41–0.84) for ICH, while the corresponding results at 18 months ranged from 0.35 (95% CI 0.22–0.51) for SVD and 0.61 (95% CI 0.44–0.75) for LAD (Figure 3). The differences between subtypes or between time points were not statistically significant.

There were impairments in terms of z-score <0 for the global measures MoCA and global z for the entire study population. MoCA z-scores were −1.18 (95% CI −1.33 to −1.02) and −1.15 (95% CI −1.31 to −0.99) at 3 and 18 months, respectively (Figure 3). The global scores were found to be impaired in terms of z-score <0 for all stroke subtypes at 3 and 18 months (Figure 3). All cognitive domains except perceptual-motor function were found to be impaired in terms of z-score <0 for the entire study population, and memory was found to be most severely impaired with a z-score of −0.85 (95% CI −0.97 to −0.73) and −0.85 (95% CI −0.97 to −0.72) at 3 and 18 months, respectively. For almost all stroke subtypes, all the cognitive domains except for perceptual-motor function were found to be impaired in terms of z-score <0 at both time points (Figure 3).

### Course of Cognition and Differences Between Stroke Subtypes

Executive function and language were found to be impaired in terms of z-score <0 in the entire stroke population at 3 months but had improved from 3 to 18 months (Figures 2C,E). Perceptual-motor function was normal in the entire stroke population at 3 months but declined from 3 to 18 months (Figure 2F). Among ICH patients, language was impaired in terms of z-score <0 at 3 months and normalized at 18 months (Figure 3E), and for LAD patients, perceptual-motor function was normal at 3 months but declined from 3 to 18 months (Figure 3F). Differences between stroke subtypes were found for attention, with impairment in terms of z-score <0 for LAD and CE but not for SVD and UD (Figure 3B).

The results were substantially the same for sensitivity analyses for unadjusted analyses; analyses excluding participants deceased at 18 months (*n* = 21) adjusted for age, education and sex; analyses excluding participants with pre-stroke dementia defined as pre-stroke GDS of 4–7 (*n* = 23) adjusted for age, education and sex; analyses adjusted for age, education, sex, pre-stroke mRS, and NIHSS combined; and analyses adjusted for age, education, sex and location of symptoms for those categorized with right or left symptom location, combined (Supplementary Figures S4–S13). The exceptions were that no statistically significant differences were found between stroke subtypes regarding attention in the analyses with the exclusion of pre-stroke dementia and for the analyses adjusted for age, education, sex, pre-stroke mRS, and NIHSS combined. In addition, for executive function for the entire stroke population, the improvement did not reach statistical significance for the analyses adjusted for age, education, sex, pre-stroke mRS, and NIHSS combined and for the analyses adjusted for age, education, sex and location of symptoms combined. Also, the improvement in language for the entire stroke population and in patients with ICH did not reach statistical significance for the analyses adjusted age, education, sex and location of symptoms combined. The numbers of participants for the different stroke subtypes included in the analyses are shown in Supplementary Table S3.

## Discussion

In this descriptive study of stroke survivors accessible for cognitive assessment, we demonstrated a high probability for PSCI at 3- and 18-months post-stroke. Impairments in global cognitive measures and several cognitive domains were identified for the entire stroke population and for almost all stroke subtypes after 3 and 18 months. Executive function and language improved for the entire stroke population, and, after categorizing the sample according to stroke subtypes, language normalized a long time after a stroke in ICH patients. No significant differences were identified between stroke subtypes in regard to severity of impairment, except for attention, which was impaired for cortical strokes but not impaired in SVD.

Our results showed a high probability for PSCI according to DSM-5 criteria in the entire stroke population at 3 and 18 months, which aligns with the findings of other recent studies (2, 3). Lo et al. reported global impairment among 35–50% of stroke victims across the different stroke subtypes early after stroke, which is in accordance with our findings (17). On a group level, we found severe impairment in almost every cognitive domain. This corresponds with findings of other recent studies; however, contrary to those studies, we found memory to be the most severely impaired of the cognitive domains (12, 17). This finding continued to be significant when patients with pre-stroke dementia were excluded. One possible explanation for this could be the older ages of our study population, as Alzheimer's disease pathology is prevalent among older people and is more strongly associated with memory impairment than with cerebrovascular disease (18). Although PSCI is a prognostic factor for disability and is demanding for patients and their caregivers, it is commonly underdiagnosed. Little is known about impairment in global cognition or in specific cognitive domains and rehabilitation outcomes in these patients (48). For example, patients with memory impairment may experience challenges in regard to learning and commitment to rehabilitation programs. Moreover, working memory and attention are, among other factors, important for executive function and, thus, for the ability to regain independence in activities of daily living (49–52). As specific cognitive rehabilitation has received little attention, there is a need for randomized clinical trials focusing on rehabilitation for patients with PSCI.

The prognosis over time for global cognition and the cognitive domains is very important for patients, for caregivers, and for the healthcare system in order to personalize rehabilitation and plan for follow-up after stroke. While most studies have found deterioration of cognition over time (4–7), we found improvement between 3 and 18 months for executive function and language for the entire population and for language among ICH patients. One explanation for this could be that the sickest patients were either excluded, lost to follow-up, or unable to complete the entire test battery. Therefore, the study population comprised people who had suffered mild strokes, and the results are valid for patients with mild strokes. Furthermore, additional assessment between 3 and 18 months could have clarified whether we had missed a curve of initial improvement followed by a longer-term cognitive decline (18). Most Norwegian stroke patients receive high-quality rehabilitation, and there is also a focus on secondary prevention of new strokes (46); thus, we are unable to conclude whether the improvements in executive function and language are due to the natural course of brain regeneration or to the effect of medical treatment and/or rehabilitation. The improvement could be explained partially by hemisphere; a greater proportion of impairment in relation to left than right hemisphere strokes, as improvement in executive function and language did not reach statistical significance when controlling for location of symptoms. However, due to a certain amount of missing data for the location of symptoms, we are unable to conclude whether the loss of statistical significance is caused by the variable or by a different population.

Research on the impacts of different etiologic stroke subtypes on the prevalence and severity of PSCI could provide new insights into the underlying mechanisms for the development and course of PSCI and, thereby, on its prevention and treatment. In agreement with several studies but in contrast to others, we found better outcomes among SVD patients than among the other stroke subtypes, as attention was most impaired in relation to cortical infarcts (CE and LAD) (17, 23). We used the TOAST classification (22) to assess the etiologic subtype of ischemic stroke, which, to a small extent, reflects the severity and localization of the stroke and is known to be important for cognitive function (6, 17, 18, 22). When controlling for premorbid function and severity of the stroke, the differences between stroke subtypes diminished, indicating that our findings are partially explained by these. There was a non-significant improvement among SVD patients from 3 to 18 months, which is in contrast to findings by Mok et al., who found that severe SVD contributed to post-stroke dementia after three years (24). Our findings could be a result of too short a follow-up time. Classifying SVD by TOAST is challenging as many patients with other subtypes also have SVD, characterized by white matter hyperintensities (WMHs) seen on MRI. Using MRI provides better visualization of SVD than CT scan, and thus, routine imaging in acute stroke patients will have an impact on the TOAST classification of SVD. In addition, the risk of misclassification bias is greater when measuring SVD by TOAST instead of by WMHs as the intensity of clinical evaluation affects the misclassification bias in TOAST. However, MRI data are less available in large research studies.

The strengths of this study are its large sample size, its multicenter design, and its highly representative group of stroke patients who were assessed both early and later after suffering a stroke. However, participants with the most severe strokes were lost to follow-up or unable to complete the entire test battery and, thereby, less likely to contribute to the analyses. Therefore, the results of our study will be more valid for patients who have experienced mild strokes. Other strengths of the study are the standardization with z-scores and the use of mixed-effects logistic and linear regression models, which minimize selection bias to some extent. The study's major limitation is its lack of its own control group and normative data for Norwegian populations. Second, all domains except one are measured with only one test per domain. Third, in the analyses of stroke subtypes, there is a lack of power, especially for the smallest group, ICH. Fourth, we encountered problems evaluating the results for perceptual-motor function based on copying different figures in the two different versions of MoCA; 7.1 was used at 18 months and 7.3 at 3 months. There was probably also a ceiling effect as most patients had normal scores (14, 17).

## Conclusion

PSCI is common for all stroke subtypes, with impairment in several cognitive domains a short time as well as a long time after a stroke. We identified improvement over time for executive function and language for the entire stroke population, and language was found to be normalized a long time after a stroke among ICH patients. In regard to attention, we found better outcomes among SVD patients than among patients with cortical strokes. Increased evidence in regard to cognitive symptom profile might be important for personalizing rehabilitation, while stroke subtypes could provide new insights into underlying mechanisms. Further research is needed on pathophysiological mechanisms, prevention, and treatment as well as on relevance for rehabilitation.

## Data Availability Statement

The datasets presented in this article are not readily available because of Norwegian regulations and conditions for informed consent. Requests to access the datasets should be directed to IS, ingvild.saltvedt@ntnu.no.

## Ethics Statement

The studies involving human participants were reviewed and approved by Regional Committee for Medical and Health Research Ethics (REC Nord), UiT Norges arktiske universitet, Postboks 6050 Langnes, 9037 Tromsø. The patients/participants provided their written informed consent to participate in this study.

## Author Contributions

IS manages the Nor-COAST study and conceived the idea for the design of the present study. SA and IS were responsible for writing the present report. SA and SL planned the statistical analyses and SA performed them. ME was responsible for the work-up with the categorization according to TOAST modified. RM-K, HI-H, HE, and YS were responsible for collecting data at their respective hospitals and for performing the TOAST classification. All authors interpreted the results and read and approved the final manuscript.

## Conflict of Interest

IS and A-BK have been investigators in the drug trial Boehringer-Ingelheim 1346.0023, and A-BK has also been an investigator for Roche BN29553. The remaining authors declare that the research was conducted in the absence of any commercial or financial relationships that could be construed as a potential conflict of interest.
