# Supplementary material for: Post-stroke Cognitive Impairment—Impact of Follow-Up Time and Stroke Subtype on Severity and Cognitive Profile: The Nor-COAST Study
Source: Front Neurol. 2020 Jul 17;11:699. doi: 10.3389/fneur.2020.00699 (PMC7379332; doi:10.3389/fneur.2020.00699)
Supplement: Supplementary file 1 [file Data_Sheet_1.pdf]

Supplementary Material

1 Definitions of vascular risk factors

Hypertension was defined as pre-stroke use of antihypertensive medication.

Hypercholesterolemia was defined as pre-stroke use of lipid-lowering medication or total cholesterol ≥6.2 mmol/L and/or low-density lipoprotein ≥ 4.1 mmol/L at hospital admittance for stroke (1, 2).

Diabetes mellitus was defined as a history of diabetes mellitus identified in the patient's medical records and/or pre-stroke use of antidiabetic medication and/or HbA1c ≥ 6.5% at admittance for stroke.

Coronary heart disease was defined as a history of coronary heart disease according to medical records.

Atrial fibrillation was defined as a history of permanent or paroxysmal atrial fibrillation or atrial flutter detected on an electrocardiogram and described in medical records and/or permanent or paroxysmal atrial fibrillation or atrial flutter detected on an electrocardiogram and/or telemetry during hospital stay.

Previous stroke or TIA was defined as a history of previous stroke or TIA identified in medical records.

2 TOAST classification as TOAST modified

The Trial of Org 10172 in Acute Stroke Treatment (TOAST) classification (3), used to classify etiological stroke subtypes in the present study, generates a large group in the category undetermined aetiology (UD) (4-6). The TOAST classification is conservative and may underscore clinically relevant risk factors for ischemic stroke, e.g., carotid stenosis is a risk factor even if it is under 50%, which is the limit set by the TOAST criteria for classification as large artery disease (LAD) (7). Furthermore, in regard to the classification of cardiac emboli as the etiology, atrial fibrillation is often underdiagnosed due to a brief monitoring period (48 hrs) (8).

To achieve an etiology as clinically relevant as possible for ischemic strokes, we aimed to identify the most-likely stroke etiology even in the group of the TOAST classification labelled UD. Therefore, experienced stroke physicians first applied the original TOAST criteria and classified these according to TOAST *probable* (3). The results for TOAST *probable* are shown in Figure S1; 232 (41%) ischemic strokes were classified as UD.

Based on collected data; including previous medical history, electrocardiograms, telemetry, transthoracic and transesophageal ultrasound, and information from MRI and CT scans, we performed a stepwise classification of the UD group (3, 9), first into TOAST *possible,* as described by Adams et al (3), and the details described in Figure S1; 189 (34%) ischemic strokes were still UD. Next, these UD patients were classified as TOAST *likely* (9), where participants with findings of carotid stenosis < 50% or plaques were classified as having LAD (Figure S1). In this last step, the UD group was reduced to 119 (21%). The final

TOAST classification in the present study, TOAST *modified*, was developed by merging TOAST *probable*, TOAST *possible* and TOAST *likely*.

	TOAST probable	TOAST possible	TOAST likely	TOAST modified
Large artery disease	**N=57 (10%)** Clinical symptoms of cortical or cerebellar dysfunction. Brain imaging findings of either significant (> 50%) stenosis or occlusion* Cortical or cerebellar lesion and brain stem or subcortical hemispheric infarcts of > 1.5 cm in diameter on CT or MRI	**N=16** Occlusion or stenosis > 50% contra- or ipsilaterally to the stroke lesion	**N=67** Carotid stenosis <50% or plaque	N=140 (25%)
Cardiac emboli	**N=130 (23%)** ≥ 1 Cardiac source for an embolus identified Potential large-artery atherosclerotic sources of thrombosis or embolism must have been eliminated	**N=23** Atrial fibrillation (AF) of any length detected before or during stay or strong suspicion of AF based on clinical evaluation, or findings of patent foramen ovale**, or history of previous myocardial infarction as source of cardiac embolus	N=0	N=153 (27%)
Small vessel disease	**N=128 (23%)** Lacunar syndromes†. Evidence of cerebral cortical dysfunction should be absent. CT or MRI: Normal or brain stem/subcortical hemispheric lesion < 1.5 cm Findings of cortical dysfunction or large artery pathology should be absent	**N=7** High suspicion of small vessel disease or small vessel disease detected on imaging before or during hospital stay.	N=0	N=135 (24%)
Other determined etiology	**N=17 (3.0%)** Rare causes of stroke; dissection of cerebral or cervical arteries, hypercoaguable states, hematologic disorders or non-atherosclerotic vasculopathies	N=0	N=0	N=17 (3.0%)
Undetermined etiology	**N=232 (41%)** No etiology fulfilling the strict TOAST criteria is present despite extensive vascular, cardiac and biochemical evaluation, or no cause identified but the evaluation is incomplete, or two or more competing causes of stroke are identified	N=189 (34%)	**N=119 (21%)** Embolic stroke of undetermined source, multiple possible etiologies detected or incomplete investigation and no clear etiology detected	N=119 (21%)

Figure S1. TOAST classification. First classified as TOAST *probable* based on original classification (3), then undetermined etiology (UD) of TOAST *probable* was categorized as TOAST *possible,* also based on original classification (3); those still categorized as UD were then classified as TOAST *likely* (9); finally, these were merged as TOAST *modified.*

*of a major cerebral artery or cortical branch of an artery

†most frequently being pure motor hemiparesis, pure sensory hemiparesis, ataxic hemiparesis, sensorimotor stroke, and dysarthria-clumsy hand syndrome

**on transesophageal ultrasound

3 Definition of post-stroke cognitive impairment (PSCI) according to the 5th edition of the Diagnostic and Statistical Manual of Mental Disorders criteria

Cognitive status was dichotomized into normal cognition and cognitive impairment; cognitive impairment comprised both mild and major neurocognitive disorders (NCD) and the cut-off for cognitive impairment was defined according to the cut-off for mild NCD in the 5th edition of the Diagnostic and Statistical Manual of Mental Disorders (DSM-5) criteria for mild and major neurocognitive disorders (10), as described in previous work in the Nor-COAST study (11). Five of six cognitive domains defined in DSM-5 were assessed; social cognition was not assessed. Complex attention was measured by Trail Making Test A (12); executive function by Trail Making Test B (12) and Verbal Fluency Test Letters (FAS) (13, 14); memory by Word List Memory and Recall Test (15); language by Verbal Fluency Test Category (animals) (16); and perceptual-motor function by the visuospatial/executive section of the Montreal Cognitive Assessment (MoCA), version 7.3 at 3 months and version 7.1 at 18 months (17). The probability for post-stroke cognitive impairment (PSCI), defined as mild as well as major neurocognitive disorder according to DSM-5 criteria, was based on performance on cognitive tests, and participants scoring < -1.5 SD in at least one cognitive domain were identified as having PSCI. To include participants who were unable to complete the whole test battery and to minimize bias from missing data, cognitive performance was based on MoCA scores for participants completing MoCA only and for those with incomplete cognitive testing but normal scores on completed tests.

4 4. Imputation of outcome measures

To minimize bias from excluded participants, imputation was performed as described in previous work (11) and in the following. Single items missing in the MoCA total scores were imputed by the mean of the available MoCA items for the same participant (n=1 at 3 months and n=0 at 18 months). For participants assessed by Telephone-MoCA , 8 of 30 points that could not be assessed by telephone and these 8 points were imputed by the mean of the available MoCA items for the same participant (n=20 at 3 months, where 3 had missing items in addition to the 8 points not assessed, n=25 at 18 months, where 5 had missing items in addition to the 8 points not assessed). For those participants who were able to start but not complete TMT-A (n=13 at 3 months and n=8 at 18 months) and TMT-B (n=87 at 3 months and n=53 at 18 months) due to cognitive impairment, the tests' results were set as equal to the time at the interruption of the tests, which was 300 seconds for both tests (11, 18). For global z, we imputed missing values on the domain z-scores using the mean z-scores from the other domains for the same participant at the same time point if z-scores were available for at least three of five domains (n=117 at 3 months and n=126 at 18 months). Other missing data were not imputed but treated as missing.

Supplementary Table S1. References for the normative data used for the cognitive test battery	
**Cognitive Test**	**Normative data**
Trail Making Test A (TMT-A) and B (TMT-B)	Participants ages 18–59 years or >80 years: Trail Making Test A and B: Normative data stratified by age and education (19)<br><br>Participants ags 60–79 years: Age-, Sex-, and Education-Specific Norms for an Extended CERAD Neuropsychological Assessment Battery–Results From the Population-Based LIFE-Adult-Study (20)
Verbal Fluency Test Letters (FAS)	Normative data stratified by age and education for two measures of verbal fluency: FAS and animal naming (21)
Verbal Fluency Test Category (animals)	Participants ages 18–59 years or >80 years: Normative data stratified by age and education for two measures of verbal fluency: FAS and animal naming (21)<br><br>Participants ages 60–79 years: Age-, Sex-, and Education-Specific Norms for an Extended CERAD Neuropsychological Assessment Battery–Results From the Population-Based LIFE-Adult-Study (20)
Word List Recall	Participants ages < 60 years: Consortium to Establish a Registry for Alzheimer's Disease (CERAD). Part V. A normative study of the neuropsychological battery (22)<br><br>Participants ages 60–79 years: Age-, Sex-, and Education-Specific Norms for an Extended CERAD Neuropsychological Assessment Battery–Results From the Population-Based LIFE-Adult-Study (20)<br><br>Participants ages > 80 years: CERAD-NP Battery: Age-, gender- and education-specific reference values for selected subtests. Results of the German Study on Ageing, Cognition and Dementia in Primary Care Patients (AgeCoDe) (23)
Montreal Cognitive Assessment (MoCA)	Montreal Cognitive Assessment: Normative data from a large Swedish population-based cohort (24)

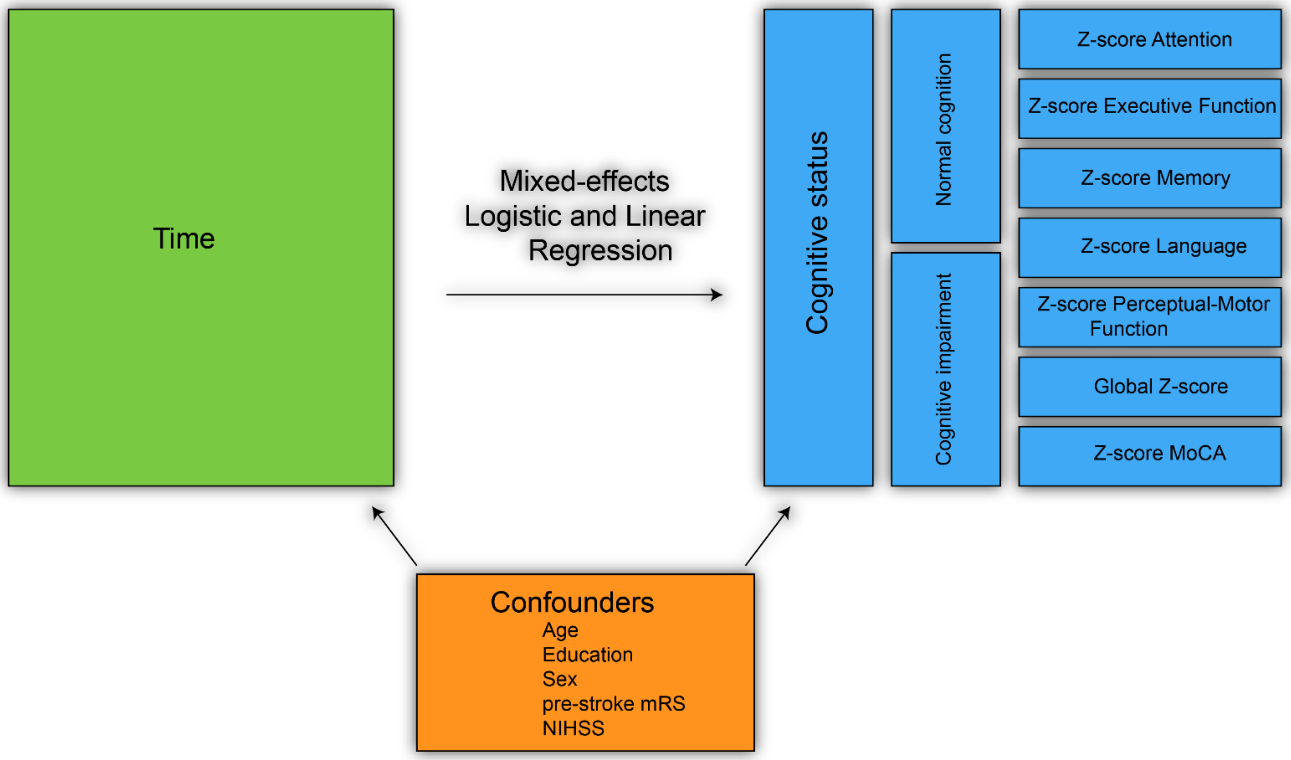

Figure S2. Illustration of the mixed-effects logistic and linear regression for model 1

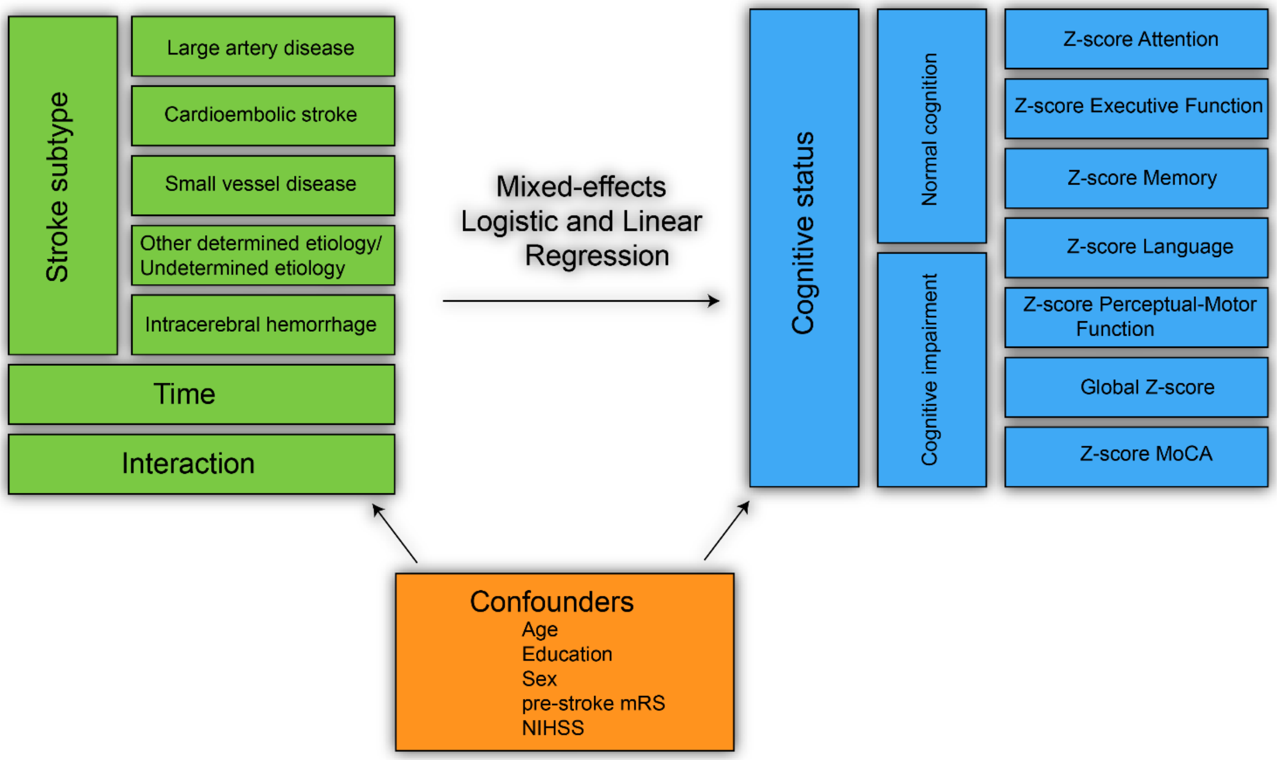

Figure S3. Illustration of the mixed-effects logistic and linear regression for model 2

Table S2. Participants' performance on the cognitive domains

	3 months			18 months		
	**N**	**Mean z-score (SD)**	**n with z<-1.5 (%)**	**N**	**Mean z-score (SD)**	**n with z<-1.5 (%)**
**Attention**	548	-0.99 (2.9)	124 (23)	440	-0.57 (2.4)	68 (15)
**Executive function**	543	-0.69 (1.5)	122 (22)	436	-0.45 (1.4)	85 (19)
**Memory**	479	-0.87 (1.4)	148 (31)	353	-0.76 (1.3)	94 (27)
**Language**	468	-0.64 (1.2)	101 (22)	328	-0.38 (1.4)	65 (20)
**Perceptual-motor function**	568	0.058 (1.1)	64 (11)	468	-0.14 (1.3)	73 (16)
**Global z**	544	-0.63 (1.2)	99 (18)	438	-0.46 (1.1)	55 (13)
**MoCA**	588	-1.2 (2.1)	205 (35)	493	-0.95 (2.1)	153 (31)

SD=Standard Deviation, MoCA=Montreal Cognitive Assessment

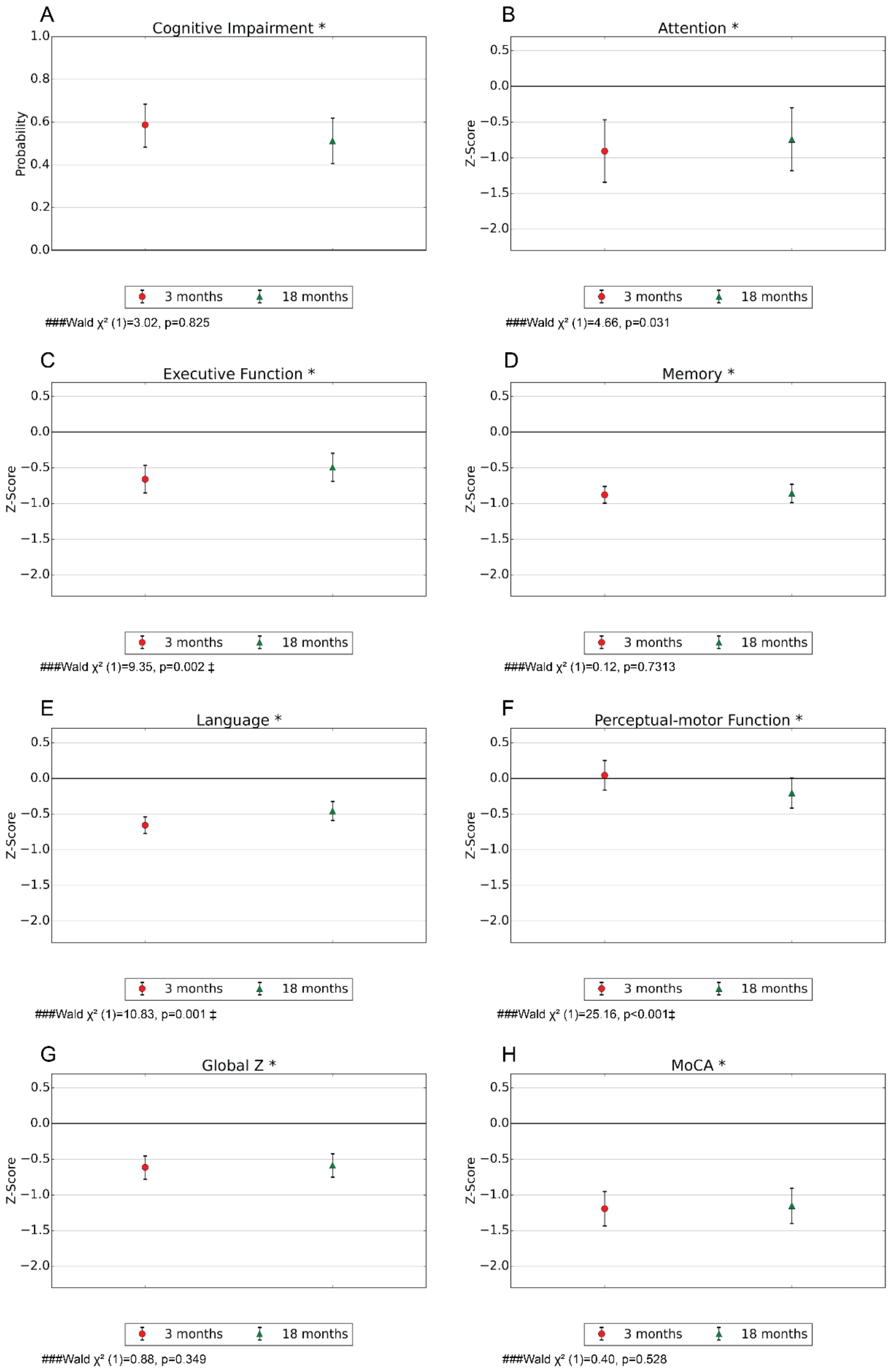

Figure S4. Sensitivity analyses without adjustment: probability for cognitive impairment according to DSM-5 criteria and mean z-scores for the cognitive domains with 95% confidence intervals at 3 and 18 months post-stroke for model 1

MoCA = Montreal Cognitive Assessment

*unadjusted analysis

Wald $\chi^2(1)$ = Wald χ^2 with one degree of freedom; test of whether there is an effect of time

‡ = $p<0.01$

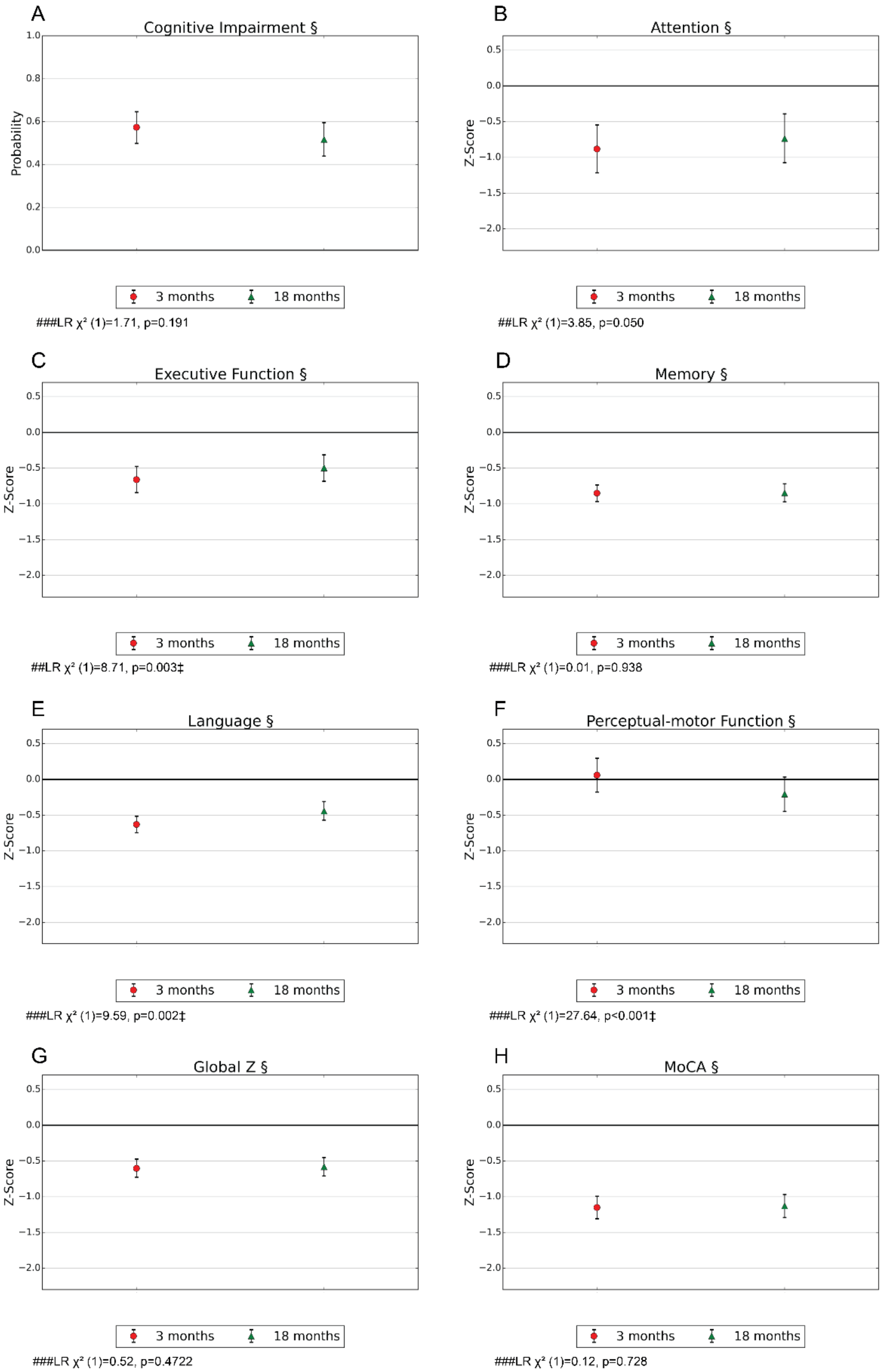

Figure S5. Sensitivity analyses with exclusion of participants deceased at 18 months: probability for cognitive impairment according to DSM-5 criteria and mean z-scores for the cognitive domains with 95% confidence intervals at 3 and 18 months post-stroke for model 1 adjusted for age, education, and sex

MoCA = Montreal Cognitive Assessment

§exclusion of participants deceased at 18 months, adjusted for age, education and sex

LR $\chi^2(1)$ = Likelihood ratio test model 1 vs a model with only age, education and sex as confounders, with one degree of freedom; hypothesis test of whether there is an effect of time

‡ = $p<0.01$

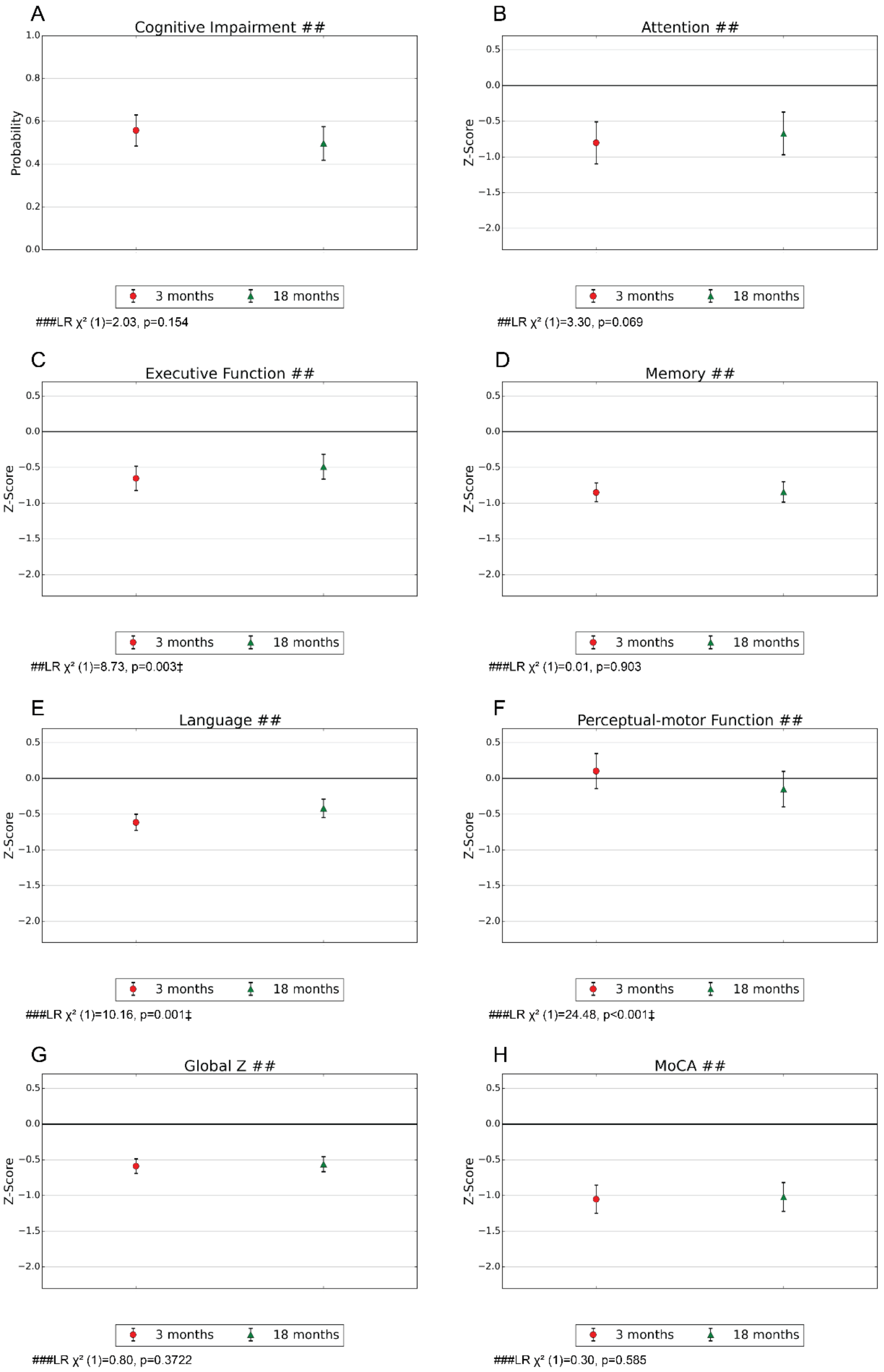

Figure S6. Sensitivity analyses with exclusion of participants with pre-stroke dementia: probability for cognitive impairment according to DSM-5 criteria and mean z-scores for the cognitive domains with 95% confidence intervals at 3 and 18 months post-stroke for model 1 for analyses adjusted for age, education, and sex

MoCA = Montreal Cognitive Assessment

exclusion of participants with pre-stroke dementia, defined as pre-stroke Global Deterioration Scale 4-7, adjusted for age, education, and sex

LR $\chi^2(1)$ =Likelihood ratio test model 1 vs a model with only age, education, and sex as confounders, with one degree of freedom; hypothesis test of whether there is an effect of time

‡ = $p<0.01$

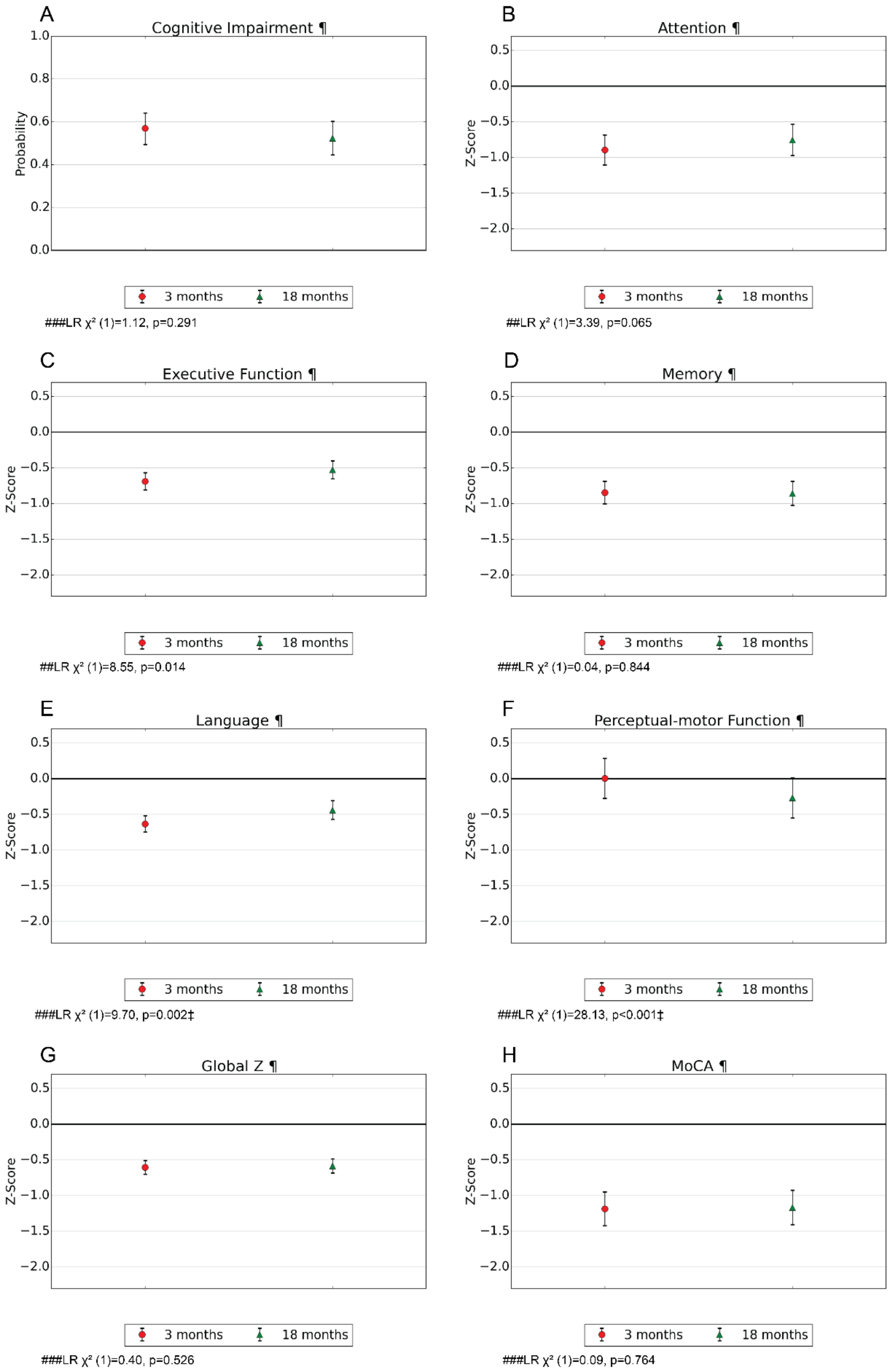

Figure S7. Sensitivity analyses with adjustment for age, education, sex, pre-stroke mRS, NIHSS: probability for cognitive impairment according to DSM-5 criteria and mean z-scores for the cognitive domains with 95% confidence intervals at 3 and 18 months post-stroke for model 1

MoCA = Montreal Cognitive Assessment

¶ adjusted for age, education and sex, pre-stroke modified Rankin Scale (mRS), National Institutes of Health Stroke Scale (NIHSS)

LR $\chi^2(1)$ = Likelihood ratio test model 1 vs a model with only age, education,sex, pre-stroke mRS, and NIHSS as confounders, with one degree of freedom; hypothesis test of whether there is an effect of time

‡ = $p<0.01$

B

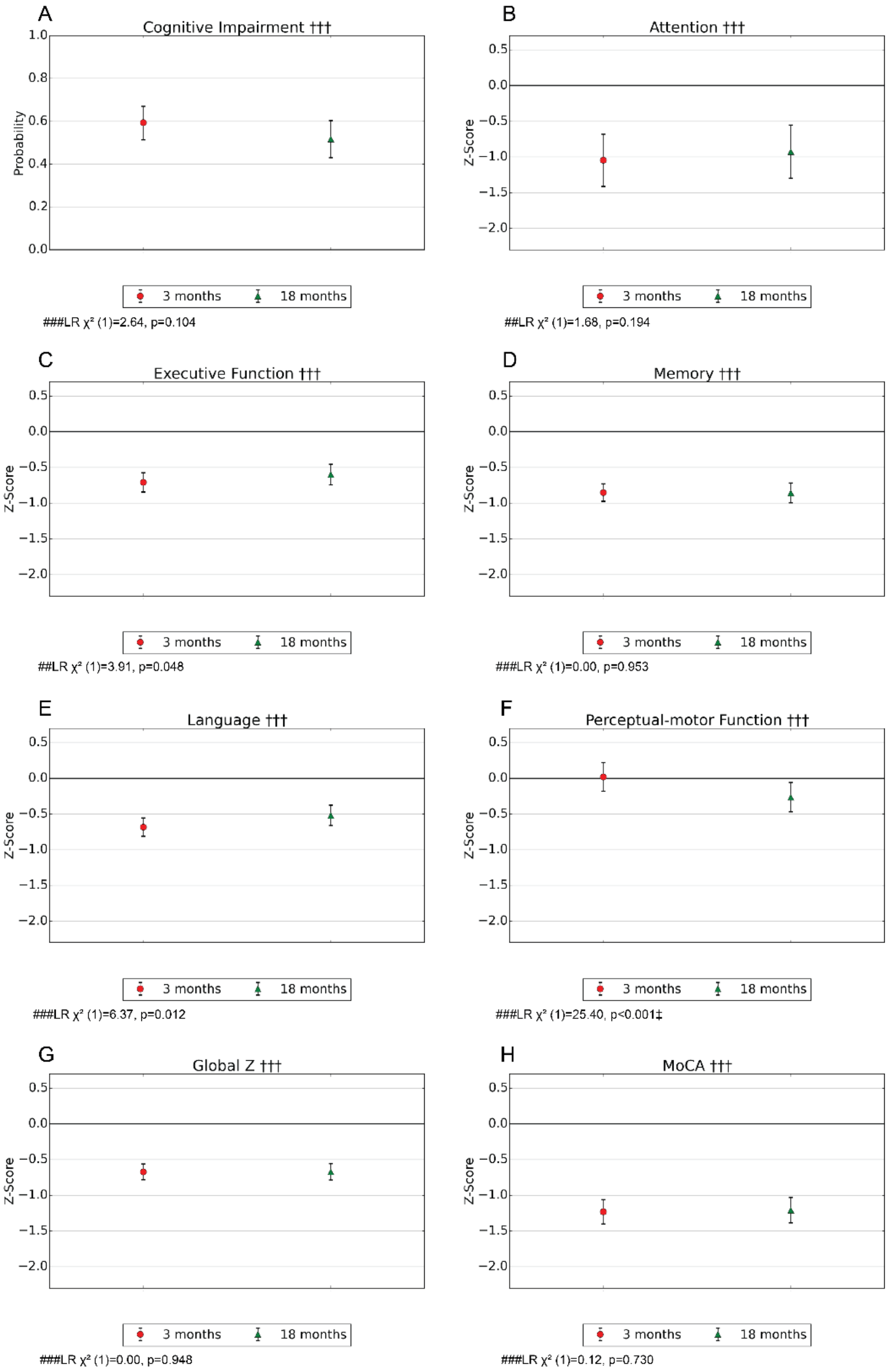

Figure S8. Sensitivity analyses with adjustment for age, education, sex, and location of symptoms: probability for cognitive impairment according to DSM-5 criteria and mean z-scores for the cognitive domains with 95% confidence intervals at 3 and 18 months post-stroke for model 1

MoCA = Montreal Cognitive Assessment

††† adjusted for age, education, sex, and location of symptoms

LR $\chi^2(1)$ = Likelihood ratio test model 1 vs a model with only age, education, sex and location of symptoms as confounders, with one degree of freedom; hypothesis test of whether there is an effect of time

‡ = $p<0.01$

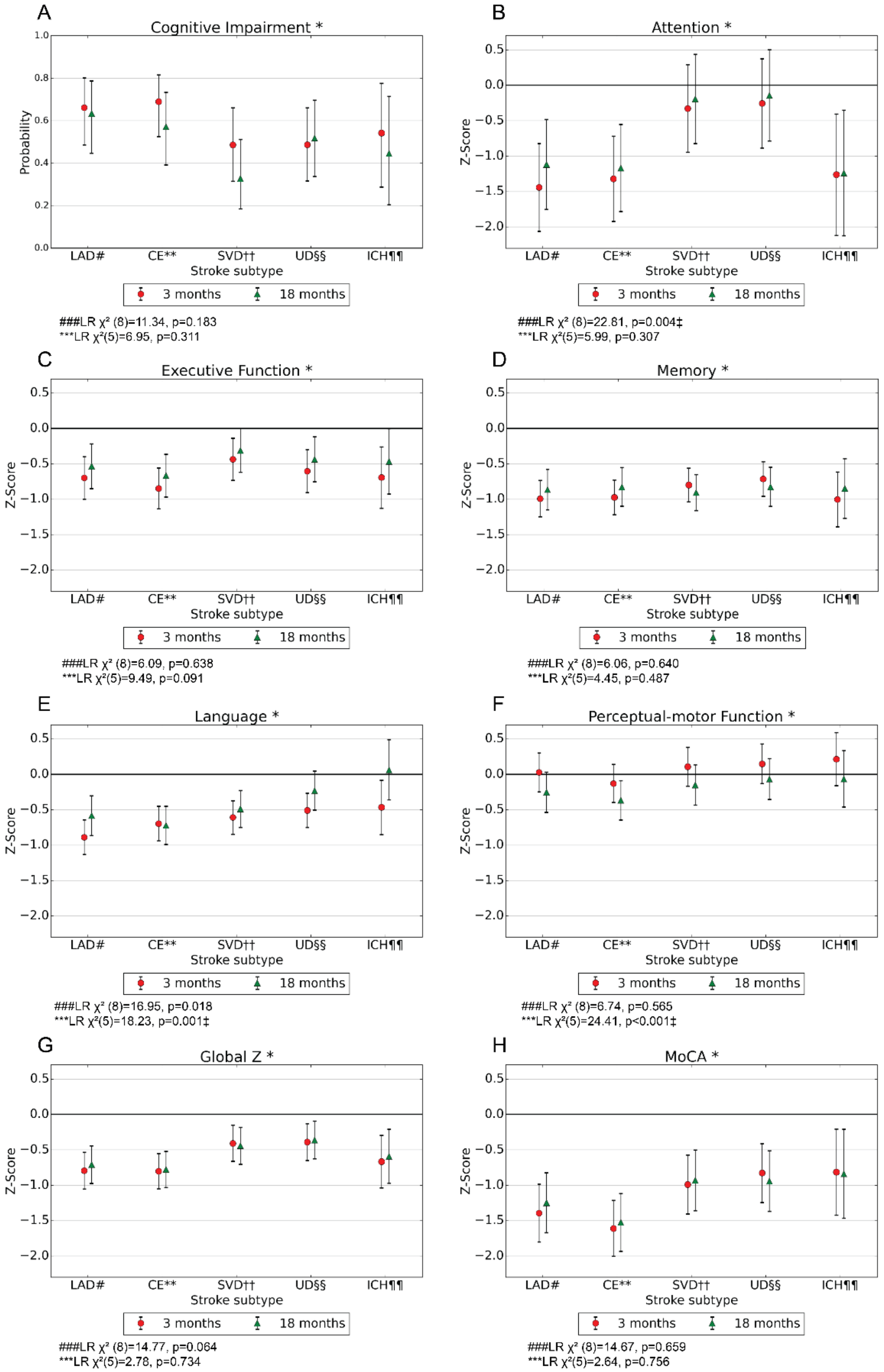

Figure S9. Sensitivity analyses without adjustment: probability for PSCI according to DSM-5 criteria and mean z-scores for the cognitive domains with 95% confidence intervals at 3 and 18 months post-stroke for model 2

MoCA = Montreal Cognitive Assessment

*unadjusted analysis

#LAD = Large artery disease

**CE = Cardiac emboli

††SVD = Small vessel disease

§§UD = Undetermined and other determined strokes

¶¶ICH = Intracerebral hemorrhage

LR $\chi^2(8)$ = Likelihood ratio test model 1 vs model 2 with 8 degrees of freedom; hypothesis test of whether there is an effect of stroke subtype

*** LR $\chi^2(5)$ =Likelihood ratio test model 2 vs model 3 with 5 degrees of freedom; hypothesis test of whether there is an effect of time for at least one stroke subtype

‡ = p<0.01

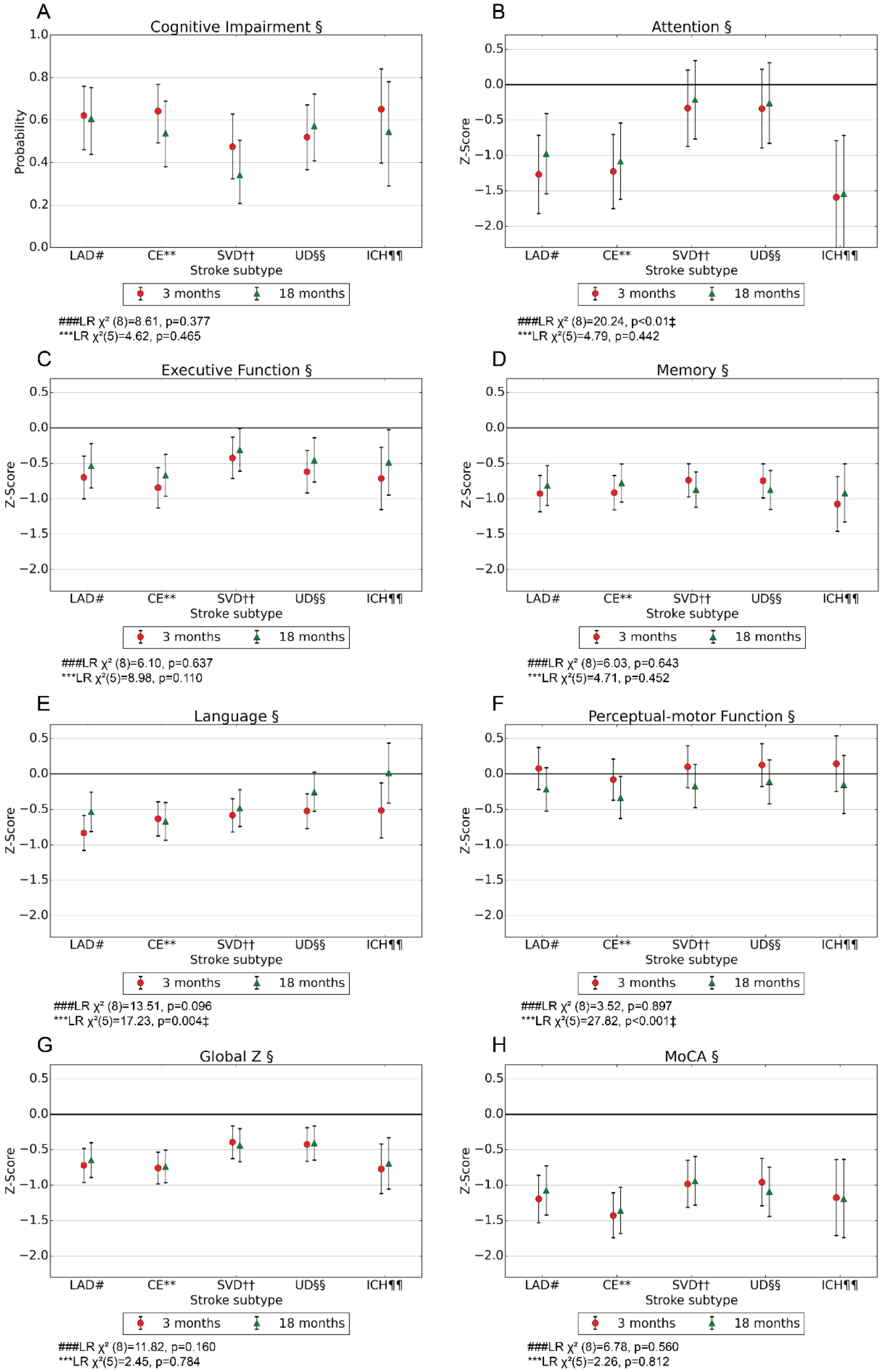

Figure S10. Sensitivity analyses with exclusion of participants deceased at 18 months: probability for cognitive impairment according to DSM-5 criteria and mean z-scores for the cognitive domains with 95% confidence intervals at 3 and 18 months post-stroke for model 2 for analyses adjusted for age, education, and sex

MoCA = Montreal Cognitive Assessment

§exclusion of participants deceased at 18 months, adjusted for age, education, and sex

#LAD = Large artery disease

**CE = Cardiac emboli

††SVD = Small vessel disease

§§UD = Undetermined- and other determined strokes

¶¶ICH = Intracerebral hemorrhage

LR $\chi^2(8)$ = Likelihood ratio test model 1 vs model 2 with 8 degrees of freedom; hypothesis test of whether there is an effect of stroke subtype

*** LR $\chi^2(5)$ = Likelihood ratio test model 2 vs model 3 with 5 degrees of freedom; hypothesis test of whether there is an effect of time for at least one stroke subtype

‡ = p<0.01

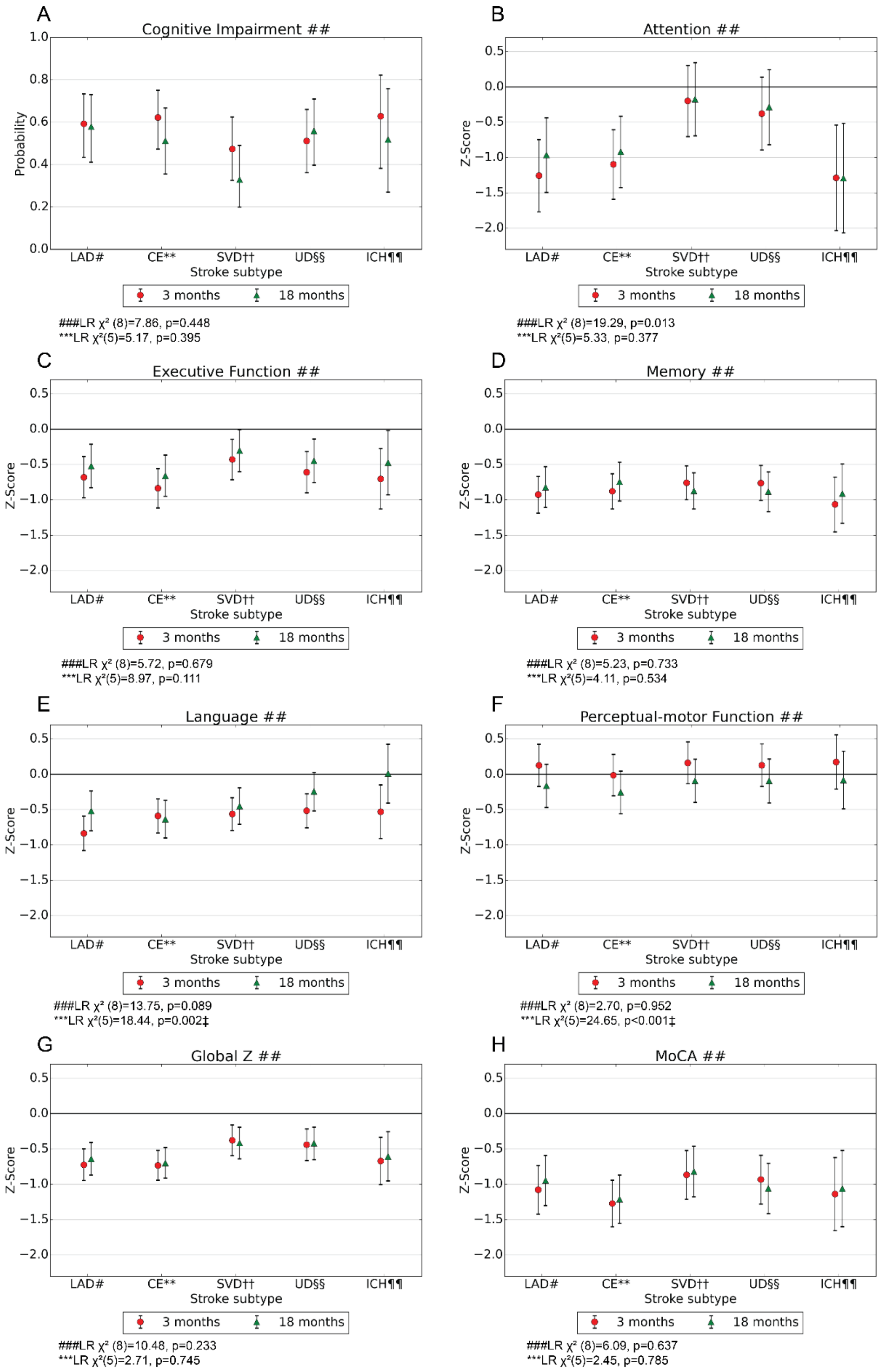

Figure S11. Sensitivity analyses with exclusion of participants with pre-stroke dementia: probability for cognitive impairment according to DSM-5 criteria and mean z-scores for the cognitive domains with 95% confidence intervals at 3 and 18 months post-stroke for model 2 for analyses adjusted for age, education, and sex

MoCA = Montreal Cognitive Assessment

exclusion of participants with pre-stroke dementia, defined as pre-stroke Global Deterioration Scale 4–7, adjusted for age, education, and sex

#LAD = Large artery disease

**CE = Cardiac emboli

††SVD = Small vessel disease

§§UD = Undetermined and other determined strokes

¶¶ICH = Intracerebral hemorrhage

LR $\chi^2(8)$ =Likelihood ratio test model 1 vs model 2 with 8 degrees of freedom; hypothesis test of whether there is an effect of stroke subtype

*** LR $\chi^2(5)$ = Likelihood ratio test model 2 vs model 3 with 5 degrees of freedom; hypothesis test of whether there is an effect of time for at least one stroke subtype

‡ = p<0.01

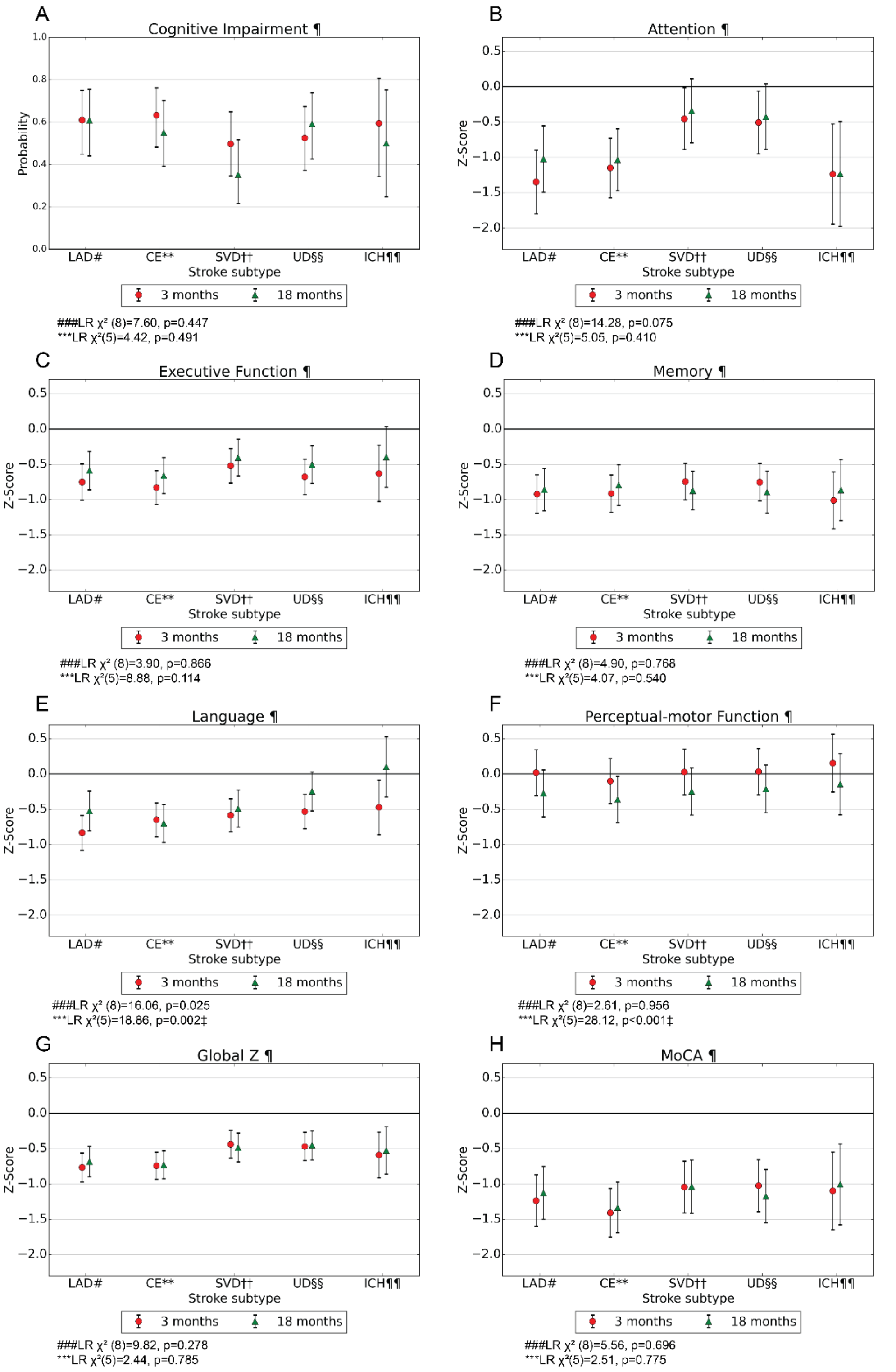

Figure S12. Sensitivity analyses with adjustment adjusted for age, education, sex, pre-stroke mRS and NIHSS: Probability for cognitive impairment according to DSM-5 criteria and mean z-scores for the cognitive domains with 95% confidence intervals at 3 and 18 months post-stroke for model 2

MoCA = Montreal Cognitive Assessment

¶ adjusted for age, education and sex, pre-stroke modified Rankin Scale (mRS), and National Institutes of Health Stroke Scale (NIHSS)

#LAD = Large artery disease

**CE = Cardiac emboli

††SVD = Small vessel disease

§§UD = Undetermined and other determined strokes

¶¶ICH = Intracerebral hemorrhage

LR $\chi^2(8)$ = Likelihood ratio test model 1 vs model 2 with 8 degrees of freedom; hypothesis test of whether there is an effect of stroke subtype

*** LR $\chi^2(5)$ = Likelihood ratio test model 2 vs model 3 with 5 degrees of freedom; hypothesis test of whether there is an effect of time for at least one stroke subtype

‡ = $p<0.01$

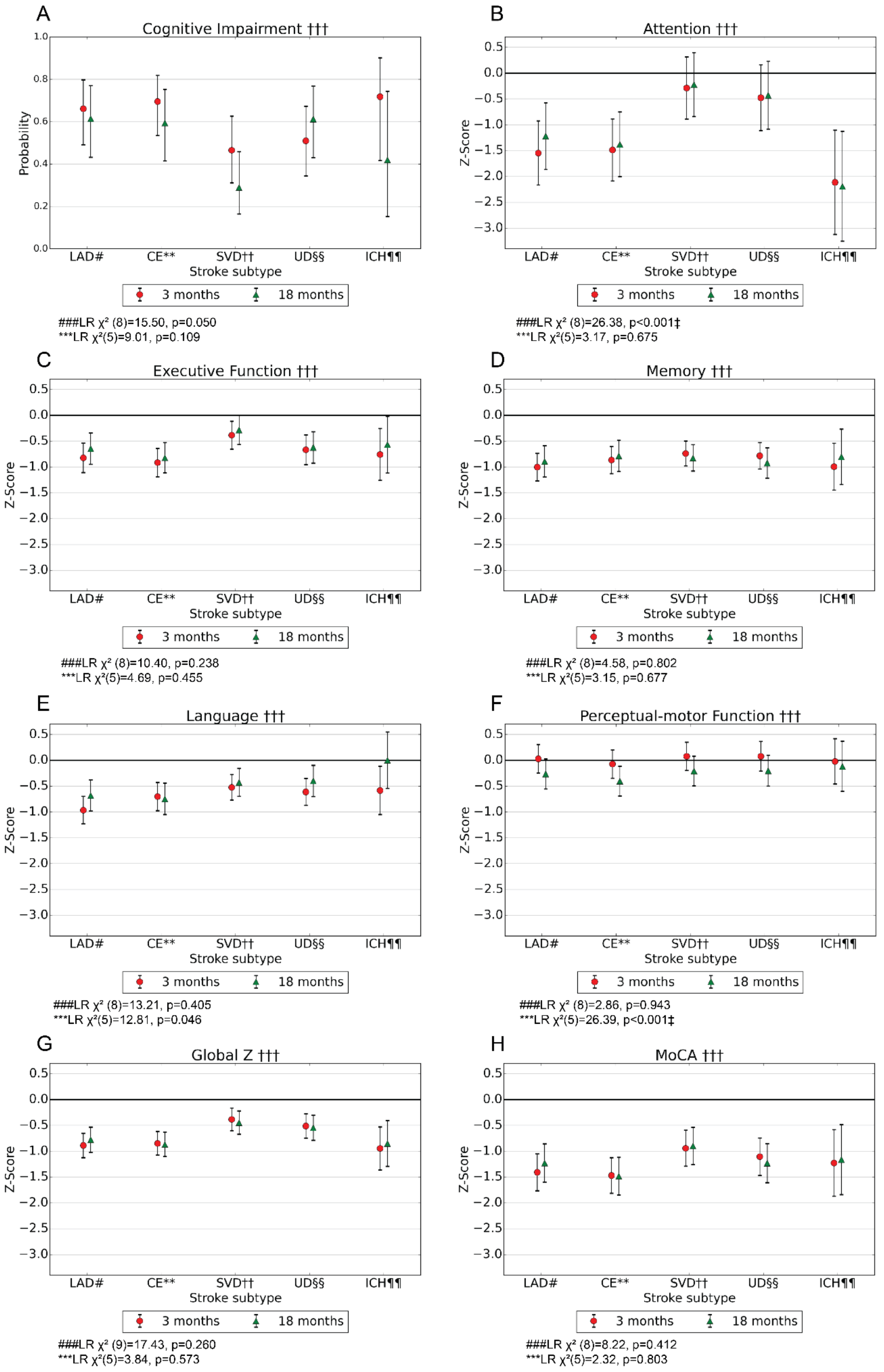

Figure S13. Sensitivity analyses with adjustment for age, education, sex and location of symptoms: probability for cognitive impairment according to DSM-5 criteria and mean z-scores for the cognitive domains with 95% confidence intervals at 3 and 18 months post-stroke for model 2

MoCA = Montreal Cognitive Assessment

††† adjusted for age, education and sex, and location of symptoms

#LAD = Large artery disease

**CE = Cardiac emboli

††SVD = Small vessel disease

§§UD = Undetermined and other determined strokes

¶¶ICH = Intracerebral hemorrhage

LR $\chi^2(8)$ = Likelihood ratio test model 1 vs model 2 with 8 degrees of freedom; hypothesis test of whether there is an effect of stroke subtype

*** LR $\chi^2(5)$ = Likelihood ratio test model 2 vs model 3 with 5 degrees of freedom; hypothesis test of whether there is an effect of time for at least one stroke subtype

‡ = $p<0.01$

**Table S3. Numbers of participants of different stroke subtypes included in the analyses**								
			**Stroke subtype**					
			**LAD**	**CE**	**SVD**	**UD**	**ICH**	**Total**
**Probability for cognitive impairment**	Unadjusted analyses and analyses adjusted for age, education, and sex	3 months	130	147	129	131	52	589
		18 months	110	121	110	110	45	496
	Analyses adjusted for age, education, and sex, exclusion of deceased (n=20)	3 months	125	142	126	126	50	569
		18 months	110	120	110	110	45	495
	Analyses adjusted for age, education, and sex, exclusion of pre-stroke dementia (n=20)	3 months	124	140	126	128	51	569
		18 months	107	116	108	109	44	484
	Analyses adjusted for age, education, sex, pre-stroke mRS, and NIHSS	3 months	125	143	126	129	50	573
		18 months	108	119	108	108	42	485
	Analyses adjusted for age, education, sex, and location of symptoms	3 months	110	120	116	107	35	488
		18 months	93	97	98	90	29	407
**Attention**	Unadjusted analyses and analyses adjusted for age, education, sex, pre-stroke mRS, and NIHSS	3 months	124	136	126	118	50	548
		18 months	93	108	104	96	39	440

	Analyses adjusted for age, education, and sex, exclusion of deceased (n=18)	3 months	112	133	123	114	48	530
		18 months	93	108	104	96	39	440
	Analyses adjusted for age, education, and sex, exclusion of pre-stroke dementia (n=13)	3 months	114	132	124	116	49	535
		18 months	92	106	103	96	39	436
	Analyses adjusted for age, education, sex, pre-stroke mRS, and NIHSS	3 months	115	132	123	116	48	534
		18 months	91	107	102	94	38	432
	Analyses adjusted for age, education, sex, and location of symptoms	3 months	99	113	113	96	33	454
		18 months	76	85	92	79	24	356
**Executive function**	Unadjusted analyses and analyses adjusted for age, education, and sex	3 months	117	133	125	119	49	543
		18 months	93	106	103	96	38	436
	Analyses adjusted for age, education, and sex, exclusion of deceased (n=17)	3 months	111	131	122	115	47	526
		18 months	93	106	103	96	38	436
	Analyses adjusted for age, education, and sex, exclusion of pre-stroke dementia (n=10)	3 months	113	130	124	117	49	533
		18 months	91	104	102	96	38	431

	Analyses adjusted for age, education, sex, pre-stroke mRS, and NIHSS	3 months	112	130	122	117	48	529
		18 months	91	105	101	94	37	428
	Analyses adjusted for age, education, sex, and location of symptoms	3 months	98	109	112	97	32	448
		18 months	76	83	91	79	22	351
**Memory**	Unadjusted analyses and analyses adjusted for age, education, and sex	3 months	99	110	114	112	44	479
		18 months	71	78	95	75	34	353
	Analyses adjusted for age, education, and sex, exclusion of deceased (n=15)	3 months	95	108	111	108	42	464
		18 months	71	78	95	75	34	353
	Analyses adjusted for age, education, and sex, exclusion of pre-stroke dementia (n=10)	3 months	95	107	113	111	43	469
		18 months	70	77	93	75	34	349
	Analyses adjusted for age, education, sex, pre-stroke mRS, and NIHSS	3 months	96	107	112	110	42	467
		18 months	69	77	93	73	33	345
	Analyses adjusted for age, education, sex, and location of symptoms	3 months	86	81	100	89	28	384
		18 months	59	58	73	56	17	263

**Language**	Unadjusted analyses and analyses adjusted for age, education, and sex	3 months	105	104	111	106	42	468
		18 months	69	77	81	70	31	328
	Analyses adjusted for age, education, and sex, exclusion of deceased (n=15)	3 months	101	102	108	102	40	453
		18 months	69	77	81	70	31	328
	Analyses adjusted for age, education, and sex, exclusion of pre-stroke dementia (n=9)	3 months	101	101	110	105	42	459
		18 months	67	76	80	70	31	324
	Analyses adjusted for age, education, sex, pre-stroke mRS, and NIHSS	3 months	100	103	108	104	41	456
		18 months	67	76	79	68	30	320
	Analyses adjusted for age, education, sex and location of symptoms	3 months	86	81	100	89	28	384
		18 months	59	58	73	56	17	263
**Perceptual-motor function**	Unadjusted analyses and analyses adjusted for age, education, and sex	3 months	125	140	129	123	51	568
		18 months	104	113	108	103	40	468
	Analyses adjusted for age, education, and sex, exclusion of deceased (n=20)	3 months	120	135	126	118	49	548
		18 months	104	113	108	103	40	468

	Analyses adjusted for age, education, and sex, exclusion of pre-stroke dementia (n=19)	3 months	119	133	126	121	50	549
		18 months	102	109	106	102	39	458
	Analyses adjusted for age, education, sex, pre-stroke mRS, and NIHSS	3 months	122	136	126	121	49	554
		18 months	102	111	106	101	38	458
	Analyses adjusted for age, education, sex, and location of symptoms	3 months	105	114	116	101	34	470
		18 months	87	90	96	85	24	382
**Global z**	Unadjusted analyses and analyses adjusted for age, education, and sex	3 months	117	132	126	119	50	544
		18 months	93	107	104	96	38	438
	Analyses adjusted for age, education, and sex, exclusion of deceased (n=16)	3 months	112	130	123	115	48	528
		18 months	93	107	104	96	38	438
	Analyses adjusted for age, education, and sex, exclusion of pre-stroke dementia (n=11)	3 months	113	129	125	117	49	533
		18 months	91	105	103	96	38	433
	Analyses adjusted for age, education, sex, pre-stroke mRS, and NIHSS	3 months	114	129	123	117	48	531
		18 months	91	106	102	94	37	430

	Analyses adjusted for age, education, sex, and location of symptoms	3 months	98	108	113	97	33	449
		18 months	76	84	92	79	22	353
**MoCA**	Unadjusted analyses and analyses adjusted for age, education, and sex	3 months	130	147	129	130	52	588
		18 months	109	120	110	110	44	493
	Analyses adjusted for age, education, and sex, exclusion of deceased (n=20)	3 months	125	142	126	125	50	568
		18 months	109	120	110	110	44	493
	Analyses adjusted for age, education, and sex, exclusion of pre-stroke dementia (n=20)	3 months	124	140	126	127	51	568
		18 months	106	116	108	109	43	482
	Analyses adjusted for age, education, sex, pre-stroke mRS, and NIHSS	3 months	125	143	126	128	50	572
		18 months	107	118	108	108	41	482
	Analyses adjusted for age, education, sex, and location of symptoms	3 months	110	120	116	106	35	487
		18 months	92	97	98	90	28	405

LAD = Large artery disease, CE = cardioembolic strokes, SVD = small vessel disease, UD = undetermined and other etiology, ICH = intracerebral hemorrhage, MoCA = Montreal Cognitive Assessment, mRS = modified Rankin Scale, NIHSS = National Institutes of Health Stroke Scale

5 References

1. Expert panel on detection e, and treatment of high blood, adults ci. Executive Summary of the Third Report of the National Cholesterol Education Program (NCEP) Expert Panel on Detection, Evaluation, and treatment of high blood cholesterol in adults (Adult Treatment Panel III). *JAMA* (2001) 285(19):2486-97. Epub 2001/05/23. PubMed PMID: 11368702.
2. Ihle-Hansen H, Vigen T, Ihle-Hansen H, Ronning OM, Berge T, Thommessen B, et al. Prevalence of carotid plaque in a 63- to 65-year-old Norwegian cohort from the general population: The ACE (Akershus Cardiac Examination) 1950 study. *Journal of the American Heart Association* (2018) 7(10). Epub 2018/05/10. doi: 10.1161/jaha.118.008562. PubMed PMID: 29739796; PubMed Central PMCID: PMCPMC6015330.
3. Adams HP, Jr., Bendixen BH, Kappelle LJ, Biller J, Love BB, Gordon DL, et al. Classification of subtype of acute ischemic stroke. Definitions for use in a multicenter clinical trial. TOAST–Trial of Org 10172 in Acute Stroke Treatment. *Stroke* (1993) 24(1):35-41. Epub 1993/01/01. PubMed PMID: 7678184.
4. Amarenco P, Bogousslavsky J, Caplan LR, Donnan GA, Hennerici MG. Classification of stroke subtypes. *Cerebrovasc Dis* (2009) 27(5):493-501. doi: 10.1159/000210432.
5. Chen PH, Gao S, Wang YJ, Xu AD, Li YS, Wang D. Classifying ischemic stroke, from TOAST to CISS. *CNS Neurosci Ther* (2012) 18(6):452-6. Epub 2012/01/25. doi: 10.1111/j.1755-5949.2011.00292.x. PubMed PMID: 22268862.
6. Bogiatzi C, Wannarong T, McLeod AI, Heisel M, Hackam D, Spence JD. SPARKLE (Subtypes of ischaemic stroke classification system), incorporating measurement of carotid plaque burden: A new validated tool for the classification of ischemic stroke subtypes. *Neuroepidemiology* (2014) 42(4):243-51. doi: 10.1159/000362417.
7. Bulwa Z, Gupta A. Embolic stroke of undetermined source: The role of the nonstenotic carotid plaque. *J Neurol Sci* (2017) 382:49-52. Epub 2017/11/08. doi: 10.1016/j.jns.2017.09.027. PubMed PMID: 29111018.
8. Andrade JG, Field T, Khairy P. Detection of occult atrial fibrillation in patients with embolic stroke of uncertain source: a work in progress. *Front Physiol* (2015) 6:100. Epub 2015/04/18. doi: 10.3389/fphys.2015.00100. PubMed PMID: 25883570; PubMed Central PMCID: PMCPMC4381503.
9. Michel P, Odier C, Rutgers M, Reichhart M, Maeder P, Meuli R, et al. The Acute STroke Registry and Analysis of Lausanne (ASTRAL): design and baseline analysis of an ischemic stroke registry including acute multimodal imaging. *Stroke* (2010) 41(11):2491-8. Epub 2010/10/12. doi: 10.1161/strokeaha.110.596189. PubMed PMID: 20930152.
10. American Psychiatric Association. *Diagnostic and statistical manual of mental disorders : DSM-5*. 5th ed. Washington, DC: American Psychiatric Association (2013).
11. Munthe-Kaas R, Aam S, Ihle-Hansen H, Lydersen S, Knapskog A-B, Wyller TB, et al. Impact of different methods defining post-stroke neurocognitive disorder: The Nor-COAST study. *Alzheimer's Dement* (2020) 6(1):e12000. doi: 10.1002/trc2.12000.
12. Reitan RM. Validity of the Trail Making Test as an indicator of organic brain damage. *Percept Mot Skills* (1958) 8:271-6.
13. Bechtoldt HP, Benton AL, Fogel ML. An application of factor analysis in neuropsychology. *Psychol Record* (1962) 12(2):147-56.
14. Fogel ML. The Gerstmann syndrome and the parietal symptom-complex. *Psychol Record* (1962) 12(1):85-99.
15. Morris JC, Mohs RC, Rogers H, Fillenbaum G, Heyman A. Consortium to establish a registry for Alzheimer's disease (CERAD) clinical and neuropsychological assessment of Alzheimer's disease. *Psychopharmacol Bull* (1988) 24(4):641-52. Epub 1988/01/01. PubMed PMID: 3249766.

16. Morris JC, Heyman A, Mohs RC, Hughes JP, van Belle G, Fillenbaum G, et al. The consortium to establish a registry for Alzheimer’s disease (CERAD). Part I. Clinical and neuropsychological assessment of Alzheimer’s disease. *Neurology* (1989) 39(9):1159-65.
17. Nasreddine ZS, Phillips NA, Bedirian V, Charbonneau S, Whitehead V, Collin I, et al. The Montreal Cognitive Assessment, MoCA: a brief screening tool for mild cognitive impairment. *J Am Geriatr Soc* (2005) 53(4):695-9. Epub 2005/04/09. doi: 10.1111/j.1532-5415.2005.53221.x. PubMed PMID: 15817019.
18. Teuschl Y, Ihle-Hansen H, Matz K, Dachenhausen A, Ratajczak P, Tuomilehto J, et al. Multidomain intervention for the prevention of cognitive decline after stroke – a pooled patient-level data analysis. *Eur J Neurol* (2018) 25(9):1182-8. doi: 10.1111/ene.13684.
19. Tombaugh TN. Trail Making Test A and B: Normative data stratified by age and education. *Arch Clin Neuropsychol* (2004) 19(2):203-14. doi: 10.1016/s0887-6177(03)00039-8. PubMed PMID: WOS:000220314900005.
20. Luck T, Pabst A, Rodriguez FS, Schroeter ML, Witte V, Hinz A, et al. Age-, sex-, and education-specific norms for an extended CERAD Neuropsychological Assessment Battery-Results from the population-based LIFE-Adult-Study. *Neuropsychology* (2018) 32(4):461-75. Epub 2018/03/09. doi: 10.1037/neu0000440. PubMed PMID: 29517259.
21. Tombaugh TN, Kozak J, Rees L. Normative data stratified by age and education for two measures of verbal fluency: FAS and animal naming. *Arch Clin Neuropsychol* (1999) 14(2):167-77. Epub 2003/11/01. PubMed PMID: 14590600.
22. Welsh KA, Butters N, Mohs RC, Beekly D, Edland S, Fillenbaum G, et al. The Consortium to Establish a Registry for Alzheimer's Disease (CERAD). Part V. A normative study of the neuropsychological battery. *Neurology* (1994) 44(4):609-14. Epub 1994/04/01. PubMed PMID: 8164812.
23. Luck T, Riedel-Heller SG, Wiese B, Stein J, Weyerer S, Werle J, et al. [CERAD-NP battery: Age-, gender- and education-specific reference values for selected subtests. Results of the German Study on Ageing, Cognition and Dementia in Primary Care Patients (AgeCoDe)]. *Z Gerontol Geriatr* (2009) 42(5):372-84. doi: 10.1007/s00391-009-0031-y. PubMed PMID: 19639242.
24. Borland E, Nagga K, Nilsson PM, Minthon L, Nilsson ED, Palmqvist S. The Montreal Cognitive Assessment: Normative Data from a Large Swedish Population-Based Cohort. *J Alzheimers Dis* (2017) 59(3):893-901. Epub 2017/07/13. doi: 10.3233/jad-170203. PubMed PMID: 28697562; PubMed Central PMCID: PMCPMC5545909.
